# Paramagnetic salt and agarose recipes for phantoms with desired T1 and T2 values for low‐field MRI

**DOI:** 10.1002/nbm.5281

**Published:** 2024-11-17

**Authors:** Kalina V. Jordanova, Carla C. Fraenza, Michele N. Martin, Ye Tian, Sheng Shen, Christopher E. Vaughn, Kevin J. Walsh, Casey Walsh, Charlotte R. Sappo, Stephen E. Ogier, Megan E. Poorman, Rui P. Teixeira, William A. Grissom, Krishna S. Nayak, Matthew S. Rosen, Andrew G. Webb, Steven G. Greenbaum, Velencia J. Witherspoon, Kathryn E. Keenan

**Affiliations:** ^1^ Physical Measurement Laboratory National Institute of Standards and Technology Boulder Colorado USA; ^2^ Department of Physics and Astronomy, Hunter College City University of New York New York New York USA; ^3^ Ming Hsieh Department of Electrical and Computer Engineering University of Southern California Los Angeles California USA; ^4^ Athinoula A. Martinos Center for Biomedical Imaging Massachusetts General Hospital and Harvard Medical School Boston Massachusetts USA; ^5^ Vanderbilt University Institute of Imaging Science Vanderbilt University Nashville Tennessee USA; ^6^ Department of Biomedical Engineering Vanderbilt University Nashville Tennessee USA; ^7^ Department of Mechanical and Aerospace Engineering The Ohio State University Columbus Ohio USA; ^8^ Department of Physics University of Colorado Boulder Boulder Colorado USA; ^9^ Hyperfine, Inc Guilford Connecticut USA; ^10^ Radiology and Radiological Sciences Vanderbilt University Medical Center Nashville Tennessee USA; ^11^ Department of Physics Harvard University Cambridge Massachusetts USA; ^12^ C.J. Gorter MRI Center, Department of Radiology Leiden University Medical Center Leiden The Netherlands; ^13^ Section on Quantitative Imaging and Tissue Sciences Eunice Kennedy Shriver National Institute of Child Health and Human Development, National Institutes of Health Bethesda Maryland USA; ^14^ Department of Biomedical Engineering Tulane University New Orleans Louisiana USA

**Keywords:** low‐field, neurological, phantoms, quantitative, relaxation

## Abstract

Tissue‐mimicking reference phantoms are indispensable for the development and optimization of magnetic resonance (MR) measurement sequences. Phantoms have greatest utility when they mimic the MR signals arising from tissue physiology; however, many of the properties underlying these signals, including tissue relaxation characteristics, can vary as a function of magnetic field strength. There has been renewed interest in magnetic resonance imaging (MRI) at field strengths less than 1 T, and phantoms developed for higher field strengths may not be physiologically relevant at these lower fields. This work focuses on developing materials with specific relaxation properties for lower magnetic field strengths. Specifically, we developed recipes that can be used to create synthetic samples for target nuclear magnetic resonance relaxation values for fields between 0.0065 and 0.55 T. $T_{1}$ and $T_{2}$ mixing models for agarose‐based gels doped with a paramagnetic salt (one of CuSO_4_, GdCl_3_, MnCl_2_, or NiCl_2_) were created using relaxation measurements of synthetic gel samples at 0.0065, 0.064, and 0.55 T. Measurements were evaluated for variability with respect to measurement repeatability and changing synthesis protocol or laboratory temperature. The mixing models were used to identify formulations of agarose and salt composition to approximately mimic the relaxation times of five neurological tissues (blood, cerebrospinal fluid, fat, gray matter, and white matter) at 0.0065, 0.0475, 0.05, 0.064, and 0.55 T. These mimic sample formulations were measured at each field strength. Of these samples, the GdCl_3_ and NiCl_2_ measurements were closest to the target tissue relaxation times. The GdCl_3_ or NiCl_2_ mixing model recipes are recommended for creating target relaxation samples below 0.55 T. This work can help development of MRI methods and applications for low‐field systems and applications.

## Graphical abstract

Recipes that use agarose mixed with one of four paramagnetic salts are developed (NiCl_2_ shown here), which can be used to create synthetic samples for field strengths below 0.55 T. The recipes' utility is demonstrated by targeting relaxation properties of five neurological tissues for fields ranging between 0.0065 and 0.55 T.

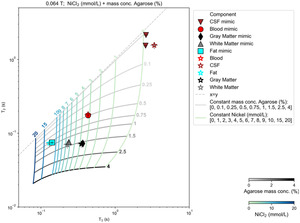

**Abbreviations**
CSF: cerebrospinal fluid
EDTA: editic acid
FFC: fast field cycling
GM: gray matter
MR: magnetic resonance
MRI: magnetic resonance imaging
NMR: nuclear magnetic resonance
PN: part number
qMRI: quantitative MRI
SI: supplemental information
WM: white matter

## 1 INTRODUCTION

There is renewed interest in magnetic resonance imaging (MRI) at field strengths less than 1 T due to the potential for lower cost, greater portability, quieter operation, improved accessibility, improved implant safety, reduced susceptibility artifacts, and reduced specific absorption rate compared to conventional systems (1.5 and 3 T).^1^, ^2^, ^3^ Many scanners are in development that operate at fields below 1 T.^4^, ^5^, ^6^, ^7^, ^8^, ^9^, ^10^, ^11^, ^12^, ^13^ Additionally, there are many commercial products available or under development for lower field strengths, including 0.064 T (Swoop, Hyperfine, Guilford, CT, USA), 0.066 T (Promaxo Inc., Oakland CA, USA), 0.345 T (MRIdian Linac, ViewRay, Mountain View, CA, USA), 0.4 T (Magnifico Open, Esaote, Genoa, Italy), 0.5 T (Evry, Synaptive Medical Inc., Toronto, Canada), and 0.55 T (MAGNETOM Free. Max, Siemens Healthineers, Erlangen, Germany).

Tissue‐mimicking reference objects (phantoms) are indispensable for developing and optimizing MRI sequences; however, there has been limited development for lower fields. Phantoms help assess the accuracy and generalizability of imaging protocols across scanners and sites^14^ and aid in developing and translating quantitative MRI (qMRI) methods to clinical settings.^15^ Phantoms have the greatest utility when they mimic the MR signals resulting from tissue physiology. This work focuses on the relaxation properties of phantom materials. Because tissue relaxation properties vary with $B_{0}$, phantoms developed with specific relaxation characteristics for > 1 T have relaxation times that do not match tissue relaxation values at low field. Therefore, dedicated effort is needed to develop reference materials for lower fields. Developing a single phantom for all “lower” field instruments is challenging, as it would require the ability to mimic tissue relaxation over a wide range of fields. Therefore, in this work, we provide recipes to target relaxation values using a suite of easily accessible materials, with each recipe applicable to a different field strength.

Specifically, we aimed to create recipes for tissue mimics at 0.0065, 0.064, and 0.55 T. After measuring relaxation properties on a variety of samples that we prepared, we fit mixing models that correlate the concentration of agarose and a chosen paramagnetic salt to $T_{1}$ and $T_{2}$ at each field strength. While many materials may replicate relaxation properties, we used a mixture of agarose and one of four easily sourced paramagnetic salts (CuSO_4_, GdCl_3_, MnCl_2_, NiCl_2_). These components are widely available, easy to use, familiar to the MR community,^16^, ^17^, ^18^, ^19^ and the combination of agarose, and a salt provides two semi‐independent levers for modifying $T_{1}$ and $T_{2}$.^20^ We selected agarose rather than agar due to its robustness to bacterial growth and chose to use four salts to offer more options for creating reference samples in a laboratory setting. As a proof of concept, we used the resulting mixing models to create mimics of white matter (WM), gray matter (GM), fat, cerebrospinal fluid (CSF), and blood for each salt at five target field strengths (0.0065, 0.0475, 0.05, 0.064, and 0.55 T) and measured the relaxation values at each field strength.

## 2 METHODS

### 2.1 T_1_ and T_2_ mixing models

Mixing models to represent the relationship between either $T_{1}$ or $T_{2}$ and sample composition were created for mixtures of agarose with one of four salts (CuSO_4_, GdCl_3_, MnCl_2_, NiCl_2_), for three field strengths (0.0065, 0.064, 0.55 T). For each salt and field, a second‐order polynomial model was fit^21^, ^22^ relating $T_{1}$ and $T_{2}$ to salt (N) and agarose (G) concentrations:

(1) $$\frac{1}{T_{1}}=a_{1}+a_{2}G+a_{3}G^{2}+a_{4}N+a_{5}N^{2}+a_{6}\mathrm{GN}+a_{7}G^{2}N+a_{8}GN^{2}+a_{9}G^{2}N^{2}$$

(2) $$\frac{1}{T_{2}}=b_{1}+b_{2}G+b_{3}G^{2}+b_{4}N+b_{5}N^{2}+b_{6}\mathrm{GN}+b_{7}G^{2}N+b_{8}GN^{2}+b_{9}G^{2}N^{2}$$

These models were fit using $T_{1}$ and $T_{2}$ times measured from a set of test samples at each field strength (sample protocol and measurement details below). Deionized water measurements established {*a*,*b*}_1_. Then, an agarose‐only model limited to the first three terms was fit to determine {*a*,*b*}_2–3_. A salt‐only model was fit to determine {*a*,*b*}_4–5_. Finally, {*a*,*b*}_1–5_ were fixed when modeling Equations 1–2 with all terms. Coefficients were constrained to be nonnegative, and small coefficients below 1 × 10^−4^ were set to zero (no appreciable effect was observed in the fit).

To use the mixing models as recipes for creating mimic samples, agarose and salt concentrations can be calculated for target $T_{1}$ and $T_{2}$ times by inverting Equations 1–2 using Python sympy for each field strength. For this study, mimic sample compositions were calculated for five field strengths (0.0065, 0.0475, 0.05, 0.064, and 0.55 T), wherein for the 0.0475 and 0.05 T samples, the 0.064 T mixing models were used as a proxy for the models at those field strengths.

### 2.2 Sample preparation protocols

To obtain data to fit the mixing models, test samples of mixtures of agarose and a salt (Millipore‐Sigma, St. Louis, MO, USA) were identified to span a wide range of $T_{1}$ and $T_{2}$, and their relaxation properties were measured. Sample materials were chosen to be readily available and to minimize toxicity. They included CuSO_4_·5H_2_0 (part number [PN]: 209198), GdCl_3_·6H_2_0 (PN: 203289), edetic acid (EDTA) (PN: 324503), MnCl_2_·4H_2_0 (PN: 221279), NiCl_2_·6H_2_0 (PN: N6136), and agarose (PN: A6013).

Test samples were prepared by dissolving stock solutions of salt compounds in deionized water. The GdCl_3_‐EDTA stock solution was made by stirring GdCl_3_ and EDTA at a 1:2 molar ratio at 98°C for 30 min. Agarose was added to the salt solution for samples requiring a gelling agent. The solution underwent two heat cycles comprising a 30‐s interval microwave cycle until boiling and a 10‐min hotplate cycle to ensure well‐hydrated agarose.^16^ Partway through the study, this protocol was adjusted to account for evaporative loss, and additional deionized water was added to the agarose following the heat cycles. The revised protocol was used for 18 of 45 agarose test samples. The remaining agarose samples, as well as the salt‐only samples, omitted evaporative loss correction. The final mixtures were poured into vials (50 mL, 30 mL, or 10 mm nuclear magnetic resonance [NMR] tubes) pre‐rinsed with isopropyl alcohol to minimize bubble formation. Samples were prepared in large batches and divided into multiple individual vials, which were each subsequently shipped to different measurement sites (see below for measurement site details). Test sample compositions are listed in Table S1.

### 2.3 T_1_ and T_2_ measurement

Mixing models were created for each salt by fitting the models to measured sample data at 0.0065, 0.064, and 0.55 T. This work was part of a multi‐institutional effort; thus, measurements for each field strength were conducted on a different system at a different site. At 0.0065 T, measurements were made on an ultra‐low‐field scanner.^5^, ^23^ At 0.064 T, measurements were made on a Hyperfine Swoop (hardware 1.8, software rc8.5.0 and rc8.6.0, Guilford, CT, USA). At 0.55 T, measurements were made on a prototype MAGNETOM Aera XQ (Siemens Healthineers, Erlangen, Germany).

Additional measurements were acquired to validate and analyze measurement accuracy. Supplemental temperature‐controlled measurements were acquired on two variable field NMR systems: the first magnet (Resonance Research Inc. BFM‐0C, Billerica, MA, USA) was set to 0.0065 T, and the second magnet (Bruker B‐E25, Billerica, MA, USA) was set to either 0.064 or 0.55 T.

Finally, access to a fast field cycling (FFC) relaxometer (Spinmaster FFC2000 1T Relaxometer, Stelar s.r.l., Mede [PV], Italy) allowed for additional $T_{1}$ measurements to be made over a range of field strengths using only one system to compare relaxation dispersion trends for different salt and agarose mixtures.

Although the measurement protocols were not enforced to be the same between sites, all sites used a form of an inversion‐recovery sequence to measure $T_{1}$ and a spin‐echo sequence to measure $T_{2}$. Additional details for each measurement system, as well as the measurement protocols, are listed in Table S2.

### 2.4 T_1_ and T_2_ measurement evaluation metrics

Because the measurements made for this study were multi‐institutional and made on different systems, some variability in relaxation measurement is expected. To understand the magnitude of the measurement variability, relaxation time measurements were assessed for three conditions: (1) variation in *repeat* measurements made on different measurement days that were 20 days apart, (2) variation in measurements for samples made using each preparation *protocol* (with and without evaporative loss correction), and (3) variation in measurements made at different *temperatures*. Due to scan time and sample preparation limitations, not all samples could be measured for each variability condition. Specifically, *repeat* measurements were made for 15 samples at 0.55 T. *Sample‐preparation protocol*‐dependent measurements were made for seven samples that were prepared in duplicate using different protocols at 0.0065, 0.064, and 0.55 T. *Temperature*‐dependent measurements were made for 49 samples for 0.0065 T, for 10 of the 49 samples for 0.064 T, and for 16 of the 49 samples for 0.55 T. Note that temperature was not controlled in the *sample‐preparation protocol* dependence or *repeatability* measurements. To assess variability, measurements were normalized to the mean measurement for each sample, and the mean, minimum, and maximum variations were noted for each condition. Variability within ±10% of the mean measurement value was deemed to be acceptable. Note that to fit Equations 1–2, test sample measurements were limited to those that were closest to the target temperature of 20°C for each field strength. If multiple samples were measured at the same temperature and field strength, the average of their measurements was used in the mixing model fitting process.

### 2.5 Proof of concept: tissue mimics using mixing model recipes

To demonstrate the mixing models' application to producing tissue mimics, five tissues were selected for creating $T_{1}$ and $T_{2}$ sample mimics: blood, CSF, fat, GM, and WM. Literature data supplemented with $T_{1}$ ex vivo FFC measurements were used to identify target $T_{1}$ and $T_{2}$ relaxation times for each tissue at five field strengths. This involved fitting relaxation dispersion models to tissue‐specific relaxation data and interpolating the target values at the relevant field strengths. Details about the data and dispersion models can be found in Figures S1–S2 and Tables S3–S4.

Once target relaxation times were identified for each tissue, each salt's mixing model was used to calculate agarose and salt concentrations that could serve as a mimic for the target $T_{1}$ and $T_{2}$. Because the agarose concentrations for the CSF mimics were very small, which can be difficult to synthesize, deionized water was included as an additional potential CSF mimic. For targets lying entirely outside the $T_{1}$‐$T_{2}$ sample space, mimic samples were calculated to target $T_{1}$ or $T_{2}$ separately.

Mimic samples were created using the evaporative‐corrected protocol described previously, and relaxation times were measured for five fields of interest. These measurements were made using the systems described above for the three mixing model field strengths (0.0065, 0.064, and 0.55 T), and for two additional field strengths using two different systems (0.0475 and 0.05 T). At 0.0475 T, the measurement system was a Sigwa open bi‐planar permanent magnet (Sigwa MRI, Boston, MA, USA). At 0.05 T, the system was a point‐of‐care permanent magnet‐based Halbach array.^24^ For 0.0475 and 0.05 T, the mimic sample compositions were calculated using the 0.064 T mixing models. These additional field measurements allowed for the assessment of how accurately mimics can be made from the mixing models when the target is at a slightly different field strength.

The percent error was calculated between the measured and target $T_{1}$ and $T_{2}$ for each tissue, salt, and field. This was used to evaluate how accurately the mimics represented tissue relaxation properties with the minimum, maximum, and average absolute errors reported for each salt and field.

## 3 RESULTS

### 3.1 T_1_ and T_2_ mixing models

Results of the fitted coefficients for the mixing models (Equations 1–2) for 0.0065, 0.064, and 0.55 T are given in Tables 1, 2, 3, 4. The higher order coefficients tended toward zero at 0.0065 T for all salts except MnCl_2_. For MnCl_2_, the higher order coefficients were largest for the 0.0065 T model. Relaxation measurement results for each sample used in fitting the mixing models are shown in Figures S3–S6.

**TABLE 1:** CuSO_4_ and agarose mixing model coefficients for models fit to 0.0065, 0.064, and 0.55 T $T_{1}$ and $T_{2}$ measurements.

Coeff #	0.0065 T		0.064 T		0.55 T	
	$T_{1}$ (a)	$T_{2}$ (b)	$T_{1}$ (a)	$T_{2}$ (b)	$T_{1}$ (a)	$T_{2}$ (b)
1	0.428	0.65	0.412	0.462	0.391	0.454
2	1.07	7.68	0.158	7.03	0.0324	7.47
3	0	0.0366	0.0185	0	0.00651	0.986
4	1.71	1.76	1.52	1.52	0.703	0.703
5	0.23	0.194	0.262	0.262	0.144	0.144
6	1.25	1.75	0.83	2.88	0.239	0
7	0	0	0	0	0.0182	0.146
8	0	0	0	0	0.000123	0
9	0	0	0	0	0.0212	0.98

**TABLE 2:** GdCl_3_‐EDTA and agarose mixing model coefficients for models fit to 0.0065, 0.064, and 0.55 T $T_{1}$ and $T_{2}$ measurements.

Coeff #	0.0065 T		0.064 T		0.55 T	
	$T_{1}$ (a)	$T_{2}$ (b)	$T_{1}$ (a)	$T_{2}$ (b)	$T_{1}$ (a)	$T_{2}$ (b)
1	0.428	0.65	0.412	0.462	0.391	0.454
2	1.07	7.68	0.158	7.03	0.0324	7.47
3	0	0.0366	0.0185	0	0.00651	0.986
4	18.3	18.3	15.6	15.6	8.5	8.5
5	0	0	0	0	0	0
6	0	0.0188	0	7.56	0	16.2
7	0	0	0	0.00658	0	0
8	0	7.84	0	0	0	0
9	0	0	0	0	0	0

**TABLE 3:** MnCl_2_ and agarose mixing model coefficients for models fit to 0.0065, 0.064, and 0.55 T $T_{1}$ and $T_{2}$ measurements.

Coeff #	0.0065 T		0.064 T		0.55 T	
	$T_{1}$ (a)	$T_{2}$ (b)	$T_{1}$ (a)	$T_{2}$ (b)	$T_{1}$ (a)	$T_{2}$ (b)
1	0.428	0.65	0.412	0.462	0.391	0.454
2	1.07	7.68	0.158	7.03	0.0324	7.47
3	0	0.0366	0.0185	0	0.00651	0.986
4	27.6	42.7	18.1	34.2	8.59	42.5
5	0	0	0	0	0.188	0
6	1	1	0	0	2.96	4.96
7	1	1	0	0	0.0183	0
8	1	1	0	0	0	0.125
9	1	1	0	0	0	0

**TABLE 4:** NiCl_2_ and agarose mixing model coefficients for models fit to 0.0065, 0.064, and 0.55 T $T_{1}$ and $T_{2}$ measurements.

Coeff #	0.0065 T		0.064 T		0.55 T	
	$T_{1}$ (a)	$T_{2}$ (b)	$T_{1}$ (a)	$T_{2}$ (b)	$T_{1}$ (a)	$T_{2}$ (b)
1	0.428	0.65	0.412	0.462	0.391	0.454
2	1.07	7.68	0.158	7.03	0.0324	7.47
3	0	0.0366	0.0185	0	0.00651	0.986
4	0.549	0.624	0.568	0.568	0.652	0.652
5	0.00112	0.000948	0	0	0.000977	0.000977
6	0	0.0739	0	0	0	0
7	0	0	0	0	0	0
8	0	0	0	0.00504	0	0.0525
9	0	0.0372	0	0	0	0

### 3.2 T_1_ and T_2_ measurement evaluation metrics

$T_{1}$ and $T_{2}$ measurements were measured at multiple institutions using different NMR or MRI systems at different ambient temperatures over 1.5 years, and with samples made from different sample protocols. To understand the uncertainty in these measurements, the variation in measured relaxation times under different conditions (*repeatability*, *sample‐preparation protocol* dependence, and *temperature* dependence) was assessed at 0.0065, 0.064, and 0.55 T. See the Figures S7–S13 for variation data presented in plot format.

Table 5 shows summary statistics of the mean absolute normalized error, as well as the minimum and maximum normalized error for any sample at all conditions and field strengths. For all conditions and field strengths, the mean absolute normalized error was less than 10%, indicating the variation is within the limits of acceptability, ±10%.

**TABLE 5:** Summary statistics of the mean absolute normalized error, as well as the minimum and maximum normalized error seen for any sample, for all variability conditions and field strengths. Specifically, variation due to measurement *repeatability*, *protocol* dependence, and *temperature* dependence were evaluated on a subset of samples for a subset of field strengths. Dashes indicate that the condition was not evaluated for that field strength. Conditions with normalized error above 10% are bolded, namely, for the minimum and maximum error ranges for 0.064 and 0.55 T *protocol* dependence and for 0.0065 T *temperature* dependence.

		Field (T)					
		0.0065	0.064	0.55	0.0065	0.064	0.55
		Mean absolute normalized error (%)			Minimum and maximum sample normalized error (%) (number of samples with absolute error above 10%)		
$T_{1}$	Repeatability	‐	‐	1.1	‐	‐	±1.9 (0/15)
	Protocol dependence	4.7	7.2	5.9	±5.6 (0/2)	**±17.8 (1/7)**	**−14.4 to 15.5 (1/4)**
	Temperature dependence	4.2	2.9	1.7	−9.9 to 9.0 (0/49)	−6.7 to 6.5 (0/10)	−6.9 to 6.6 (0/27)
$T_{2}$	Repeatability	‐	‐	1.7	‐	‐	±3.8 (0/15)
	Protocol dependence	5.6	4.8	9.2	±6.5 (0/2)	±8.0 (0/7)	**−14.1 to 14.3 (4/4)**
	Temperature dependence	4.3	1.3	1.4	**−19.3 to 11.3 (3/49)**	±4.1 (0/10)	−5.8 to 5.3 (0/27)

For some conditions and field strengths, the minimum and maximum normalized errors seen for a sample exceeded 10%. The number of samples with a minimum or maximum normalized error greater than 10% are indicated in Table 5. In general, very few samples had error above 10%, except for the *sample‐preparation protocol*‐dependent 0.55 T measurements for which all four samples had error larger than 10%. Note that even for this condition, the minimum and maximum normalized error was less than 15%, and many of these errors were approximately 10%. Overall, 98.2% of the $T_{1}$ measurements and 93.9% of the $T_{2}$ measurements had minimum and maximum absolute errors less than 10%.

### 3.3 Proof of concept: tissue mimics using mixing model recipes

**FIGURE 1:**
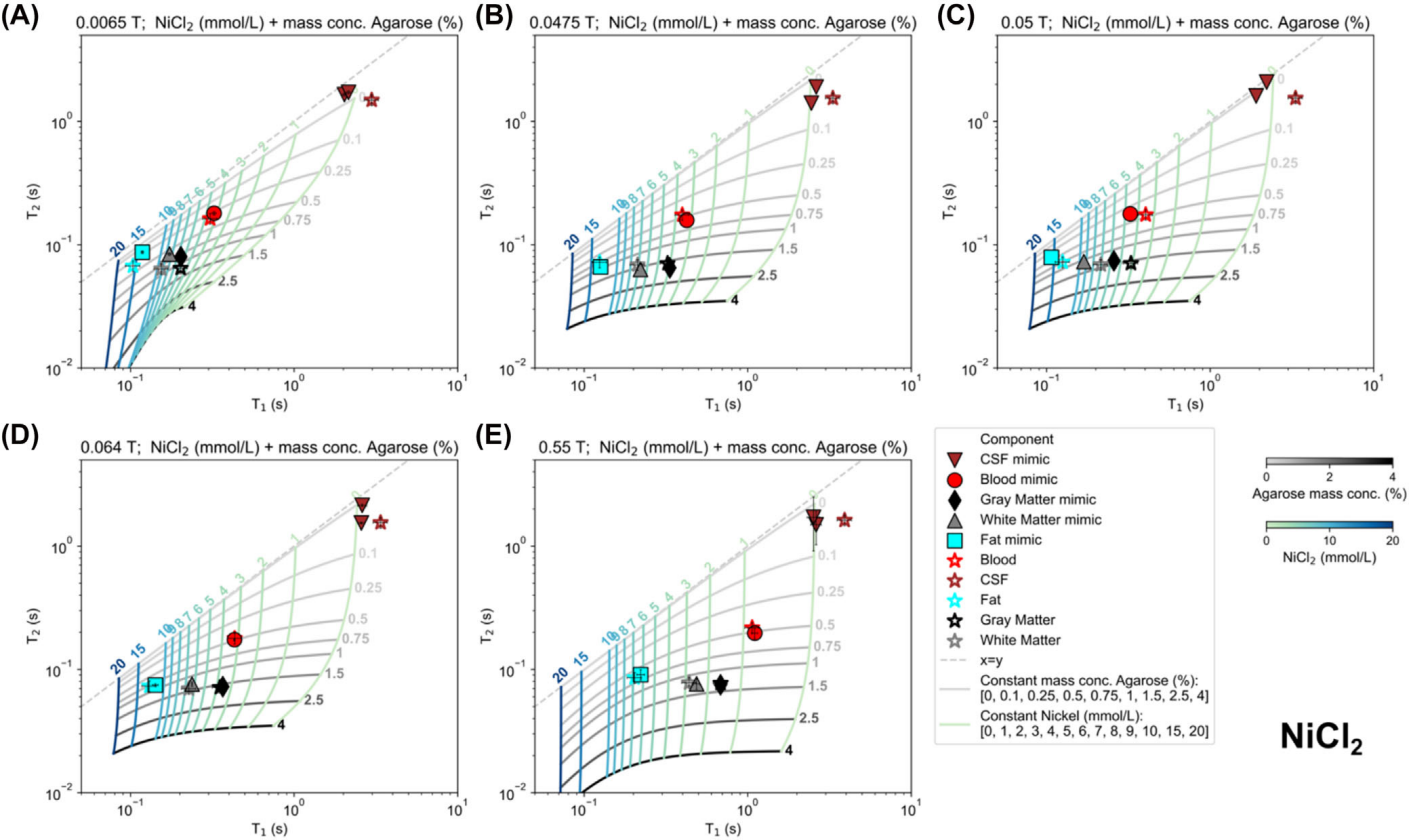
$T_{1}$ and $T_{2}$ mimic sample measurements and mixing models for NiCl_2_ with agarose for (A) 0.0065 T, (B) 0.0475 T, (C) 0.05 T, (D) 0.064 T, and (E) 0.55 T. Target tissue $T_{1}$ and $T_{2}$ times (stars; blood = red, CSF = maroon, fat = blue, GM = black, WM = gray) are shown. Each mimic measurement is shown with the same color as its tissue, and twice the standard deviation is plotted as error bars. Mixing models are displayed via constant agarose concentration lines (gray) and constant NiCl_2_ concentration lines (blue‐green). Dashed gray line represents $T_{1}=T_{2}$.

Mimic samples for five tissues were created for each field strength and each salt composition. Results comparing the measured $T_{1}$ and $T_{2}$ for each mimic to its target $T_{1}$ and $T_{2}$ for the NiCl_2_ samples are shown in Figure 1 (see the Figures S14–S16 for results for other salts).

**FIGURE 2:**
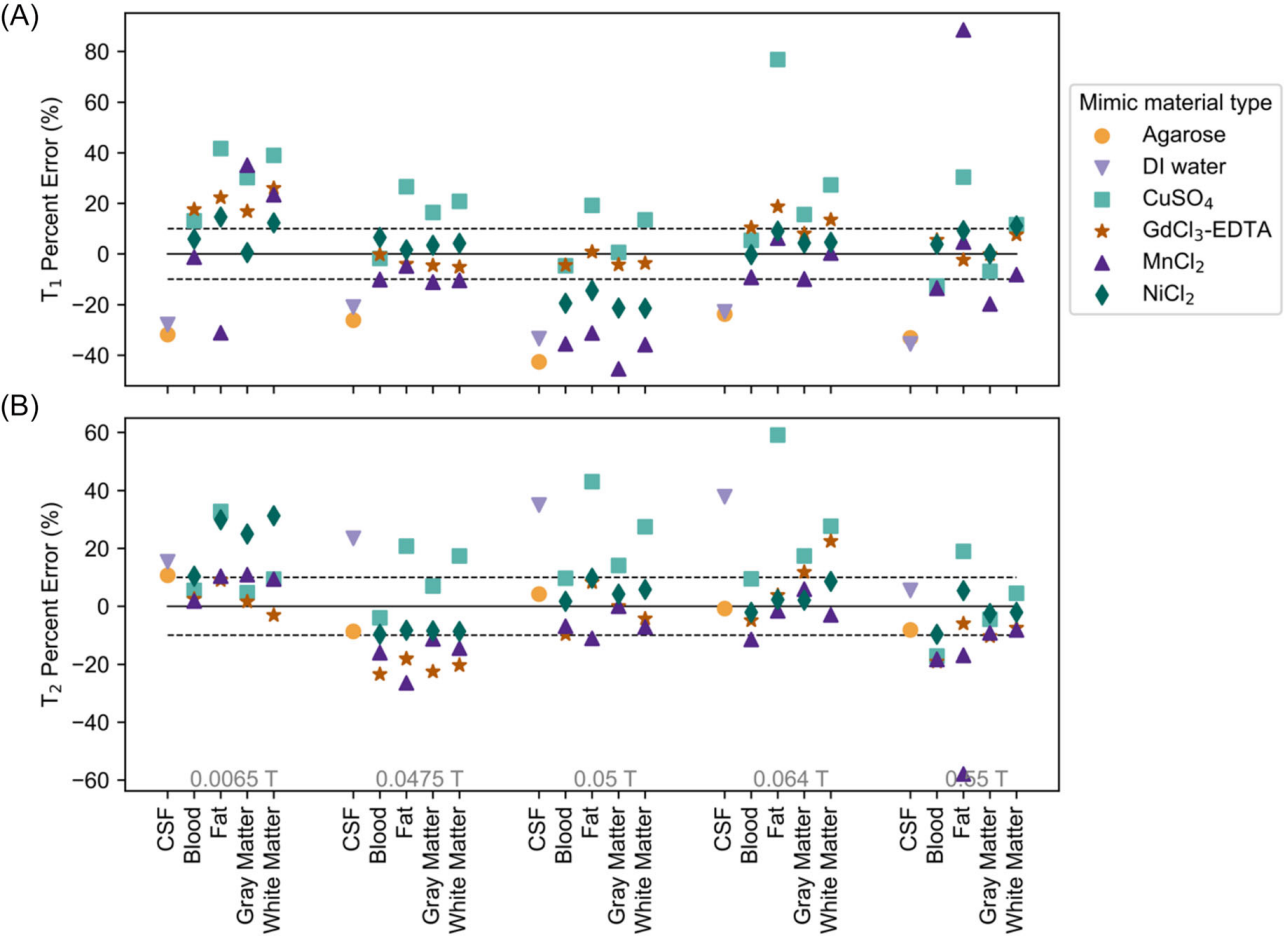
(A) $T_{1}$ and (B) $T_{2}$ mimic sample measurement error compared to the target $T_{1}$ and $T_{2}$ times, as a function of field strength, tissue, and mimic material type. Note that the NiCl_2_ errors correspond to the measurements plotted in Figure 1.

Accuracy was examined by calculating the error of each mimic's measured $T_{1}$ and $T_{2}$ from the target $T_{1}$ and $T_{2}$. The mimic sample compositions, target and measured $T_{1}$ and $T_{2}$, and mimic relaxation errors are given in Tables S5‐S8. Figure 2 shows mimic relaxation errors for $T_{1}$ and $T_{2}$, for each salt and field. Note that the NiCl_2_ errors in Figure 2 correspond to the measurements plotted in Figure 1. For all CSF mimics and for the MnCl_2_ formulated fat mimics, the relaxation error is high, because the tissue relaxation times were outside of the range of the mixing model concentration limits. Excluding CSF, which could not be mimicked by any salt, the percentages of mimics with less than 10% relaxation error (the counts of data points within the 10% confidence interval) were 35.0%, 62.5%, 42.9%, and 72.5% for CuSO_4_, GdCl_3_‐EDTA, MnCl_2_, and NiCl_2_, respectively. NiCl_2_ samples exhibited less than 10% error for the largest number of samples.

**FIGURE 3:**
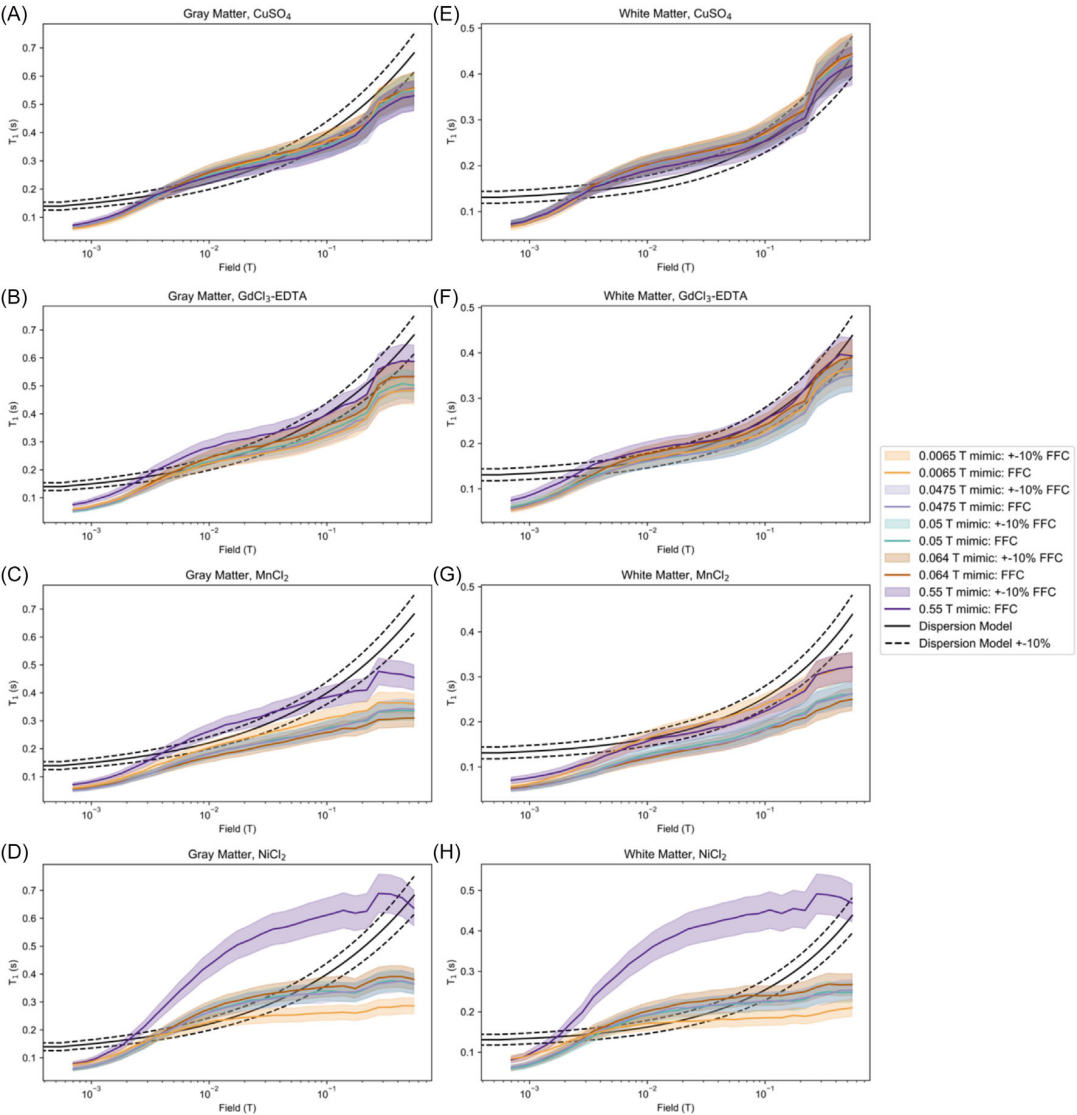
(A–D) GM and (E–H) WM mimic sample $T_{1}$ dispersion data measured on an FFC system (colored lines), along with a 10% variation of the measurements (shaded colored regions) for (A, E) CuSO_4_, (B, F) GdCl_3_‐EDTA, (C, G) MnCl_2_, and (D, H) NiCl_2_. Mimics are labeled by their target field. The $T_{1}$ dispersion model that produced the field‐specific targets for each tissue are plotted (black lines). EDTA, editic acid; FFC, fast field cycling; GM, gray matter; WM, white matter.

Finally, the $T_{1}$ of WM and GM mimics was measured on the FFC system across field strengths. Figure 3 shows $T_{1}$ measurements of the WM and GM mimics made on the FFC, as well as the WM and GM relaxation dispersion models (see the Table S4 for dispersion model details). Some of the GdCl_3_‐EDTA FFC measurements remained close to the dispersion models for a large field range: for example, the 0.0064 T mimic for WM stays within 10% of the WM $T_{1}$ targets between 0.0035 and 0.43 T, indicating that it could serve as a WM $T_{1}$ mimic for that entire field range.

## 4 DISCUSSION AND CONCLUSION

Models for mixtures of agarose and one of four paramagnetic salts were created that can be used to determine recipes for tissue mimics at low MRI field strengths of 0.0065, 0.064, and 0.55 T. $T_{1}$ and $T_{2}$ measurements for a total of 138 unique samples (52 unique test samples and 86 unique mimic samples) were measured at six institutions using eight different NMR or MRI systems at different ambient temperatures over 1.5 years. These measurements were used to create and validate the mixing models.

### 4.1 T_1_ and T_2_ mixing models

$T_{1}$ and $T_{2}$ mixing models were fit for four salt and agarose mixtures, for three fields of interest. The constraints for the coefficients of the polynomial mixing models were empirically chosen to be 0 or greater than 1 × 10^−4^ for all coefficients in this study; however, future work that defines these constraints on a per‐coefficient basis could improve the resulting mixing model fits to the measured data. Furthermore, the mixing models are sensitive to the accuracy of the measured $T_{1}$ and $T_{2}$ for each test sample. Known sources of measurement variation include variable laboratory temperature, variations in sample synthesis protocols, and variations that come from the measurement sequence parameters and fitting routines of the individual measurement sites. To minimize the variations between sites, the measurement protocols all used inversion‐recovery or spin‐echo techniques to measure $T_{1}$ and $T_{2}$, respectively. However, the fitting routines were not consistent across all sites; for some systems, the team did not have access to the raw data, only the resulting quantitative map. A potential improvement to this study would be centralized data processing. To assess the impact on relaxation measurements due to the other sources, measurement variation was analyzed via repeated measurements over time, changes in sample‐preparation protocols, and variations in temperature. In general, variations in measurement were within ±10% of the measurement average. The repeat measurement variation can assess whether homogenization of the oxygen concentration occurred prior to the first measurement. The samples with repeat measurements had a mean error of less than 1.7%, with a maximum error of 3.8% for any sample (see Table 5), indicating there was sufficient time for homogenization of the samples. We note that using the same temperatures and sample protocol for all measurements could improve this study. However, as presented here, this study better replicates the expected use of the mimics, which will likely be used in various laboratory conditions by the community.

We recommend that after a sample is made using one of the recipes determined in this study, their $T_{1}$ and $T_{2}$ times are carefully measured using validated measurement methods to give reference values, before using the samples for other development and testing. These materials can be used in any format, including anthropomorphic shapes.^25^, ^26^, ^27^, ^28^, ^29^, ^30^ The dependence of relaxation times on temperature is known for many of the salts used in this study^19^, ^31^, ^32^, ^33^; thus, we recommend that temperatures be recorded for both reference measurements and test measurements of any prepared samples. Finally, relaxation properties should be measured periodically, as sample relaxation properties can change over time. Extensive stability measurements over time were outside the scope of this study; however, studies have shown stability of salt and agarose mixtures of up to 350 days.^17^

### 4.2 Proof of concept: tissue mimics using mixing model recipes

Sample compositions for four salt and agarose mixtures were determined for 0.0065, 0.0475, 0.05, 0.064, and 0.55 T that are suitable as $T_{1}$ and $T_{2}$ tissue mimics at laboratory temperatures of approximately 20°C for blood, CSF, fat, GM, and WM. A repository that provides the functionality to solve for tissue mimic compositions given target tissue relaxation times for a particular field strength has been made available online at https://github.com/usnistgov/mri-tissue-mimics.

Mimic sample relaxation times were compared to respective target relaxation times. Differences between measured and target relaxation can be due to several factors, including sample preparation techniques, systematic measurement error, and errors that propagate from the mixing models themselves when computing sample concentrations. The NiCl_2_ mimic sample measurements had the lowest magnitude of percent error compared to their target values, and GdCl_3_‐EDTA measurements were second lowest. Note that NiCl_2_ has relatively low relaxivity, and therefore, those samples are less susceptible to errors when weighing the NiCl_2_ to prepare the stock solutions. All salts failed to mimic the relaxation values of CSF due to the difference between lab temperatures (approximately 20°C) and body temperature (approximately 37°C). No sample was found to have a long enough $T_{1}$ at lab temperature to mimic CSF at body temperature. Practically, deionized water may be the best choice as a CSF mimic because it is hard to reproducibly make extremely dilute agarose gels. One way to increase the $T_{1}$ time of deionized water at laboratory temperatures is to add deuterated water, which reduces the intermolecular relaxation sink. Although effective, this solution is not practical, as it increases cost and reduces signal intensity.^31^ While individual MnCl_2_ mimics for fat $T_{1}$ and $T_{2}$ can be made, fat lies outside of the $T_{1}$‐$T_{2}$ space for MnCl_2_, and thus, MnCl_2_ is not recommended to mimic fat. We emphasize that the developed fat mimic formulation recommendations are for $T_{1}$ and $T_{2}$ values only, as they do not replicate fat's chemical shift. However, because chemical shift in absolute units (Hz) decreases with decreasing field strength, this is not a large concern for low magnetic fields.

Some CuSO_4_ and MnCl_2_ mimics had large differences between their measured $T_{1}$ and $T_{2}$ compared to the tissue $T_{1}$ and $T_{2}$ targets, which we attribute to inaccuracy within the fitted parameters for the mixing models themselves. For short $T_{1}$ and $T_{2}$, the CuSO_4_ model predicted shorter $T_{1}$ and $T_{2}$ than measured in the mimics, which indicates low predictive capability in that region. This is because the training data for CuSO_4_ measurements contain few samples in the short relaxation region. Improvements in the CuSO_4_ mixing model could improve the accuracy of future CuSO_4_ mimics. Additionally, MnCl_2_ has higher relaxivity than the other salts (especially $r_{2}$), and thus, in the short relaxation region, the MnCl_2_ mixing model exhibited a strong dependence of $T_{1}$ and $T_{2}$ on MnCl_2_ concentration. Therefore, small inaccuracies in the model or in the concentration of MnCl_2_ could result in large inaccuracies in the predicted $T_{1}$ and $T_{2}$ values. Improvements to both models could likely be made by including more samples for fitting.

The FFC $T_{1}$ data showed that while the salt and agarose curves generally do not overlap the tissue dispersion model curves, the salt and agarose concentrations can be chosen so that their curves intersect the dispersion curves at the field of their target mimic. The GdCl_3_‐EDTA curves most closely approximated the WM dispersion profile, and GdCl_3_‐EDTA is the best candidate for mimicking $T_{1}$ of WM and GM for a wide range of field strengths below 0.55 T based on the calculated percent errors of each formulation.

The mimic samples composed in this work are not meant to be perfect mimics for the calculated $T_{1}$ and $T_{2}$ times for each tissue and field. Rather, these mimics provide relaxation properties similar to tissue $T_{1}$ and $T_{2}$, using accessible materials.

### 4.3 Other considerations

A challenge of achieving tissue‐like samples is that the quantitative behavior of tissues is complex. For example, $T_{2}$ relaxation is generally multi‐exponential. However, the more complex the tissue relaxation model is, the more difficult it is to find a mimic that satisfies all facets. Additionally, previous research found that multi‐exponential $T_{2}$ measurements can vary substantially even within a tissue type.^34^ For the purpose of this study, $T_{2}$ was modeled as a mono‐exponential parameter. This simplified model makes consistency in measurements across many sites more feasible. Furthermore, this study focused on characterizing $T_{1}$ and $T_{2}$ times and did not consider other factors such as magnetization transfer, which has been shown to contribute to relaxation measurement variability.^35^ Future improvements could be made to the study by modeling and measuring more complex material behavior.

When choosing the salt to use for sample creation and transportation, the safety of handling each material should be considered. According to safety data sheets from Millipore‐Sigma, agarose and GdCl_3_‐EDTA are generally considered non‐hazardous for shipping and handling. CuSO_4_ has environmental hazards associated with it, and MnCl_2_ and NiCl_2_ are both considered toxic solids for shipping and handling; however, we did not find issues in shipping the samples made in this study due to the small concentrations used of each material. In general, these hazards should be considered when selecting sample materials.

### 4.4 Conclusion

We created mixing models that can be used to find recipes for sample composition to target specific $T_{1}$ and $T_{2}$ relaxation times at 0.0065, 0.064, and 0.55 T. We created example sample mimics for five tissue types using the mixing models and evaluated their accuracy by measuring their relaxation properties at 0.0065, 0.0475, 0.05, 0.064, and 0.55 T. This work can help both qualitative and quantitative MRI method development for low‐field systems and applications.

## CONFLICT OF INTEREST STATEMENT

This study was partially supported by a research grant from Hyperfine, Inc. Megan E. Poorman and Rui P. Teixeira are Hyperfine, Inc employees. Matthew S. Rosen is a founder and equity holder of Hyperfine, Inc.

## DISCLOSURES

Certain commercial equipment, instruments, or materials are identified in this paper in order to specify the experimental procedure adequately. Such identification is not intended to imply recommendation or endorsement by NIST, nor is it intended to imply that the materials or equipment identified are necessarily the best available for the purpose.

## Supporting information


**Figure S1** Photograph of excised brain locations. Three samples of GM were excised, and two samples of WM were excised.
**Figure S2** (a‐e) T1 and (f‐j) T2 dispersion models for (a,f) blood, (b,g) CSF, (c,h) fat, (d,i) GM, and (e,j) WM are shown in the solid line. A 10% variation of the model is shown in dashed lines, and the data used to create the fit is displayed in blue circles for the ex vivo data collected in this study and in black diamonds for the literature data. The number of literature data points are indicated in each legend. The fields of interest for this study (0.0065 T, 0.0475 T, 0.05 T, 0.064 T, 0.55 T) are indicated by vertical dotted grey lines.
**Figure S3** T1 and T2 test sample measurements and mixing models for CuSO_4_ + agarose for (a) 0.0065 T, (b) 0.064 T, (c) 0.55 T. Mixing models are displayed via constant agarose concentration lines (solid) and constant CuSO_4_ concentration lines (dashed). Dashed gray line represents T1 = T2, and twice the standard deviation is plotted as error bars.
**Figure S4** T1 and T2 test sample measurements and mixing models for GdCl_3_‐EDTA + agarose for (a) 0.0065 T, (b) 0.064 T, (c) 0.55 T. Mixing models are displayed via constant agarose concentration lines (solid) and constant GdCl_3_‐EDTA concentration lines (dashed). Dashed gray line represents T1 = T2, and twice the standard deviation is plotted as error bars.
**Figure S5** T1 and T2 test sample measurements and mixing models for MnCl_2_ + agarose for (a) 0.0065 T, (b) 0.064 T, (c) 0.55 T. Mixing models are displayed via constant agarose concentration lines (solid) and constant MnCl_2_ concentration lines (dashed). Dashed gray line represents T1 = T2, and twice the standard deviation is plotted as error bars.
**Figure S6** T1 and T2 test sample measurements and mixing models for NiCl_2_ + agarose for (a) 0.0065 T, (b) 0.064 T, (c) 0.55 T. Mixing models are displayed via constant agarose concentration lines (solid) and constant NiCl_2_ concentration lines (dashed). Dashed gray line represents T1 = T2, and twice the standard deviation is plotted as error bars.
**Figure S7** Normalized T1 (left) and T2 (right) variation between repeat measurements for 15 samples at 0.55 T. Data are normalized to the average of the mean values for each sample. The coefficient of variation is plotted as error bars. The date of measurements is indicated by the marker.
**Figure S8** Normalized T1 (left) and T2 (right) variation between protocols for two samples at 0.0065 T. Data are normalized to the average of the mean values for each sample, for each protocol. The coefficient of variation is plotted as error bars. The date of measurements is indicated in the label. Each of these samples had an agarose component, and batch 1 did not consider evaporative water losses in the synthesis protocol, whereas batch 2 did consider evaporative water loss.
**Figure S9** Normalized T1 (left) and T2 (right) variation between protocols for 7 samples at 0.064 T. Data are normalized to the average of the mean values for each sample, for each protocol. The coefficient of variation is plotted as error bars. The date of measurements is indicated in the label. Four samples had an agarose component, and batch 1 did not consider evaporative water losses in the synthesis protocol, whereas batch 2 did consider evaporative water loss. Three samples had no agarose component and evaporative water loss was not applicable for the synthesis protocol.
**Figure S10** Normalized T1 (left) and T2 (right) variation between protocols for 4 samples at 0.55 T. Data are normalized to the average of the mean values for each sample, for each protocol. The coefficient of variation is plotted as error bars. The date of measurements is indicated in the label. All samples had an agarose component, and batch 1 did not consider evaporative water losses in the synthesis protocol, whereas batch 2 did consider evaporative water loss.
**Figure S11** Normalized T1 (left) and T2 (right) variation for a range of temperatures for 49 samples at 0.0065 T. Data are normalized to the average of the mean values for each sample. The measurement temperature is indicated by the marker.
**Figure S12** Normalized T1 (left) and T2 (right) variation for a range of temperatures for 10 samples at 0.064 T. Data are normalized to the average of the mean values for each sample. The measurement temperature is indicated by the marker.
**Figure S13** Normalized T1 (left) and T2 (right) variation for a range of temperatures for 27 samples at 0.55 T. Data are normalized to the average of the mean values for each sample. The measurement temperature is indicated by the marker.
**Figure S14** T1 and T2 mimic sample measurements and mixing models for CuSO_4_ with agarose for (a) 0.0065 T, (b) 0.0475 T, (c) 0.05 T, (d) 0.064 T, (e) 0.55 T. Target tissue T1 and T2 times (stars; Blood = red, CSF = maroon, Fat = blue, GM = black, WM = gray) are shown. Each mimic measurement is shown with the same color as its tissue, and twice the standard deviation is plotted as error bars. Mixing models are displayed via constant agarose concentration lines (gray) and constant CuSO_4_ concentration lines (blue‐green). Dashed gray line represents T1=T2.
**Figure S15** T1 and T2 mimic sample measurements and mixing models for GdCl_3_‐EDTA with agarose for (a) 0.0065 T, (b) 0.0475 T, (c) 0.05 T, (d) 0.064 T, (e) 0.55 T. Target tissue T1 and T2 times (stars; Blood = red, CSF = maroon, Fat = blue, GM = black, WM = gray) are shown. Each mimic measurement is shown with the same color as its tissue, and twice the standard deviation is plotted as error bars. Mixing models are displayed via constant agarose concentration lines (gray) and constant GdCl_3_‐EDTA concentration lines (blue‐green). Dashed gray line represents T1=T2.
**Figure S16** T1 and T2 mimic sample measurements and mixing models for MnCl_2_ with agarose for (a) 0.0065 T, (b) 0.0475 T, (c) 0.05 T, (d) 0.064 T, (e) 0.55 T. Target tissue T1 and T2 times (stars; Blood = red, CSF = maroon, Fat = blue, GM = black, WM = gray) are shown. Each mimic measurement is shown with the same color as its tissue, and twice the standard deviation is plotted as error bars. Mixing models are displayed via constant agarose concentration lines (gray) and constant MnCl_2_ concentration lines (blue‐green). Dashed gray line represents T1=T2.
**Table S1** Test samples (52 total) along with the paramagnetic salt contained in each sample that were used for fitting the mixing models. Samples that required heating and had evaporative loss correction are indicated with a Y, while samples that did not have evaporative loss correction are indicated with N. Four samples (indicated by [N, Y] in the evaporative correction column) were made twice: once with evaporative loss correction, once without.
**Table S2** Relevant scan parameters for T1 and T2 measurements, for each fixed field strength and system. When multiple parameters are given, the chosen parameters were dependent on the expected T1 and T2 times of the sample being measured.
**Table S3** Model fitting initialization values and limits for each fit parameter * To achieve a fit for CSF and GM, Ci was allowed to vary down to 1x10^6^ s^−2^. ** To achieve a fit for GM, B was initialized to 10 s^−1^.
**Table S4** Fitting results for tissue dispersion models. * For blood and GM, this was allowed to range down to 1x10^6^.
**Table S5** Target tissue T1 and T2 times for CuSO_4_ and agarose tissue mimics, along with CuSO_4_ and agarose concentrations. Measurements for T1 and T2 for each mimic sample at each field strength are listed, as well as the error of the measurement from both the target relaxation times.
**Table S6** Target tissue T1 and T2 times for GdCl_3_‐EDTA and agarose tissue mimics, along with GdCl_3_‐EDTA and agarose concentrations. Measurements for T1 and T2 for each mimic sample at each field strength are listed, as well as the error of the measurement from both the target relaxation times.
**Table S7** Target tissue T1 and T2 times for MnCl_2_ and agarose tissue mimics, along with MnCl_2_ and agarose concentrations. Measurements for T1 and T2 for each mimic sample at each field strength are listed, as well as the error of the measurement from both the target relaxation times.
**Table S8** Target tissue T1 and T2 times for NiCl_2_ and agarose tissue mimics, along with NiCl_2_ and agarose concentrations. Measurements for T1 and T2 for each mimic sample at each field strength are listed, as well as the error of the measurement from both the target relaxation times.

## Data Availability

The database of gathered measurements and code that support this study will be made openly available upon publication at these links: https://doi.org/10.18434/mds2-3063 and https://github.com/usnistgov/mri-tissue-mimics.
