# Supplementary material for: Paramagnetic salt and agarose recipes for phantoms with desired T1 and T2 values for low‐field MRI
Source: NMR Biomed. 2024 Nov 17;38(1):e5281. doi: 10.1002/nbm.5281 (PMC11602269; doi:10.1002/nbm.5281)
Supplement: Supplementary file 1 — Figure S1 Photograph of excised brain locations. Three samples of GM were excised, and two samples of WM were excised. Figure S2 (a‐e) T1 and (f‐j) T2 dispersion models for (a,f) blood, (b,g) CSF, (c,h) fat, (d,i) GM, and (e,j) WM are shown in the solid line. A 10% variation of the model is shown in dashed lines, and the data used to create the fit is displayed in blue circles for the ex vivo data collected in this study and in black diamonds for the literature data. The number of literature data points are indicated in each legend. The fields of interest for this study (0.0065 T, 0.0475 T, 0.05 T, 0.064 T, 0.55 T) are indicated by vertical dotted grey lines. Figure S3 T1 and T2 test sample measurements and mixing models for CuSO4 + agarose for (a) 0.0065 T, (b) 0.064 T, (c) 0.55 T. Mixing models are displayed via constant agarose concentration lines (solid) and constant CuSO4 concentration lines (dashed). Dashed gray line represents T1 = T2, and twice the standard deviation is plotted as error bars. Figure S4 T1 and T2 test sample measurements and mixing models for GdCl3‐EDTA + agarose for (a) 0.0065 T, (b) 0.064 T, (c) 0.55 T. Mixing models are displayed via constant agarose concentration lines (solid) and constant GdCl3‐EDTA concentration lines (dashed). Dashed gray line represents T1 = T2, and twice the standard deviation is plotted as error bars. Figure S5 T1 and T2 test sample measurements and mixing models for MnCl2 + agarose for (a) 0.0065 T, (b) 0.064 T, (c) 0.55 T. Mixing models are displayed via constant agarose concentration lines (solid) and constant MnCl2 concentration lines (dashed). Dashed gray line represents T1 = T2, and twice the standard deviation is plotted as error bars. Figure S6 T1 and T2 test sample measurements and mixing models for NiCl2 + agarose for (a) 0.0065 T, (b) 0.064 T, (c) 0.55 T. Mixing models are displayed via constant agarose concentration lines (solid) and constant NiCl2 concentration lines (dashed). Dashed gray line represent [file NBM-38-e5281-s001.docx]

**Supplementary Information**

# Contents

[Contents 1](#_Toc159491024)

[Figure Captions 1](#_Toc159491025)

[Table Captions 3](#_Toc159491026)

[Methods 4](#_Toc159491027)

[Test Samples Prepared 4](#_Toc159491028)

[$\boldsymbol{T}\mathbf{1}$ and $\boldsymbol{T}\mathbf{2}$ Measurement 6](#_Toc159491029)

[NMR System: 0.0065 T – Additional Details 8](#_Toc159491030)

[MRI System: 0.0475 T – Additional Details 8](#_Toc159491031)

[MRI System: 0.064 T – Additional Details 8](#_Toc159491032)

[MRI System: 0.55 T – Additional Details 9](#_Toc159491033)

[NMR Systems: 0.0065 T, 0.064 T, 0.55 T – Additional Details 10](#_Toc159491034)

[FFC $T1$ Measurement: 0.0007 T to 0.55 T – Additional Details 10](#_Toc159491035)

[Proof of Concept: Tissue Mimics using Mixing Model Recipes 11](#_Toc159491036)

[Tissue Relaxation Data 11](#_Toc159491037)

[Dispersion Models 12](#_Toc159491038)

[Results 17](#_Toc159491039)

[$\boldsymbol{T}\mathbf{1}$ and $\boldsymbol{T}\mathbf{2}$ Mixing Models 17](#_Toc159491040)

[$\boldsymbol{T}\mathbf{1}$ and $\boldsymbol{T}\mathbf{2}$ Measurement Evaluation Metrics 21](#_Toc159491041)

[Variation in Repeatability 21](#_Toc159491042)

[Variation in Protocol-Dependence 21](#_Toc159491043)

[Variation in Temperature-Dependence 23](#_Toc159491044)

[Proof of Concept: Tissue Mimics using Mixing Model Recipes 25](#_Toc159491045)

[References 32](#_Toc159491046)

# Figure Captions

[**Figure S1** Photograph of excised brain locations. Three samples of GM were excised, and two samples of WM were excised. 12](#_Toc159491047)

[**Figure S2** (a-e) $T1$ and (f-j) $T2$ dispersion models for (a,f) blood, (b,g) CSF, (c,h) fat, (d,i) GM, and (e,j) WM are shown in the solid line. A 10% variation of the model is shown in dashed lines, and the data used to create the fit is displayed in blue circles for the ex vivo data collected in this study and in black diamonds for the literature data. The number of literature data points are indicated in each legend. The fields of interest for this study (0.0065 T, 0.0475 T, 0.05 T, 0.064 T, 0.55 T) are indicated by vertical dotted grey lines. 16](#_Toc159491048)

[**Figure S3** $T1$ and $T2$ test sample measurements and mixing models for CuSO_4_ + agarose for (a) 0.0065 T, (b) 0.064 T, (c) 0.55 T. Mixing models are displayed via constant agarose concentration lines (solid) and constant CuSO_4_ concentration lines (dashed). Dashed gray line represents T1=T2, and twice the standard deviation is plotted as error bars. 17](#_Toc159491049)

[**Figure S4** $T1$ and $T2$ test sample measurements and mixing models for GdCl_3_-EDTA + agarose for (a) 0.0065 T, (b) 0.064 T, (c) 0.55 T. Mixing models are displayed via constant agarose concentration lines (solid) and constant GdCl_3_-EDTA concentration lines (dashed). Dashed gray line represents T1=T2, and twice the standard deviation is plotted as error bars. 18](#_Toc159491050)

[**Figure S5** $T1$ and $T2$ test sample measurements and mixing models for MnCl_2_ + agarose for (a) 0.0065 T, (b) 0.064 T, (c) 0.55 T. Mixing models are displayed via constant agarose concentration lines (solid) and constant MnCl_2_ concentration lines (dashed). Dashed gray line represents T1=T2, and twice the standard deviation is plotted as error bars. 19](#_Toc159491051)

[**Figure S6** $T1$ and $T2$ test sample measurements and mixing models for NiCl_2_ + agarose for (a) 0.0065 T, (b) 0.064 T, (c) 0.55 T. Mixing models are displayed via constant agarose concentration lines (solid) and constant NiCl_2_ concentration lines (dashed). Dashed gray line represents T1=T2, and twice the standard deviation is plotted as error bars. 20](#_Toc159491052)

[**Figure S7** Normalized $T1$ (left) and $T2$ (right) variation between repeat measurements for 15 samples at 0.55 T. Data are normalized to the average of the mean values for each sample. The coefficient of variation is plotted as error bars. The date of measurements is indicated by the marker. 21](#_Toc159491053)

[**Figure S8** Normalized $T1$ (left) and $T2$ (right) variation between protocols for two samples at 0.0065 T. Data are normalized to the average of the mean values for each sample, for each protocol. The coefficient of variation is plotted as error bars. The date of measurements is indicated in the label. Each of these samples had an agarose component, and batch 1 did not consider evaporative water losses in the synthesis protocol, whereas batch 2 did consider evaporative water loss. 21](#_Toc159491054)

[**Figure S9** Normalized $T1$ (left) and $T2$ (right) variation between protocols for 7 samples at 0.064 T. Data are normalized to the average of the mean values for each sample, for each protocol. The coefficient of variation is plotted as error bars. The date of measurements is indicated in the label. Four samples had an agarose component, and batch 1 did not consider evaporative water losses in the synthesis protocol, whereas batch 2 did consider evaporative water loss. Three samples had no agarose component and evaporative water loss was not applicable for the synthesis protocol. 22](#_Toc159491055)

[**Figure S10** Normalized $T1$ (left) and $T2$ (right) variation between protocols for 4 samples at 0.55 T. Data are normalized to the average of the mean values for each sample, for each protocol. The coefficient of variation is plotted as error bars. The date of measurements is indicated in the label. All samples had an agarose component, and batch 1 did not consider evaporative water losses in the synthesis protocol, whereas batch 2 did consider evaporative water loss. 22](#_Toc159491056)

[**Figure S11** Normalized $T1$ (left) and $T2$ (right) variation for a range of temperatures for 49 samples at 0.0065 T. Data are normalized to the average of the mean values for each sample. The measurement temperature is indicated by the marker. 23](#_Toc159491057)

[**Figure S12** Normalized $T1$ (left) and $T2$ (right) variation for a range of temperatures for 10 samples at 0.064 T. Data are normalized to the average of the mean values for each sample. The measurement temperature is indicated by the marker. 24](#_Toc159491058)

[**Figure S13** Normalized $T1$ (left) and $T2$ (right) variation for a range of temperatures for 27 samples at 0.55 T. Data are normalized to the average of the mean values for each sample. The measurement temperature is indicated by the marker. 24](#_Toc159491059)

[**Figure S14** $T1$ and $T2$ mimic sample measurements and mixing models for CuSO_4_ with agarose for (a) 0.0065 T, (b) 0.0475 T, (c) 0.05 T, (d) 0.064 T, (e) 0.55 T. Target tissue $T1$ and $T2$ times (stars; Blood=red, CSF=maroon, Fat=blue, GM=black, WM=gray) are shown. Each mimic measurement is shown with the same color as its tissue, and twice the standard deviation is plotted as error bars. Mixing models are displayed via constant agarose concentration lines (gray) and constant CuSO_4_ concentration lines (blue-green). Dashed gray line represents $T1=T2$. 25](#_Toc159491060)

[**Figure S15** $T1$ and $T2$ mimic sample measurements and mixing models for GdCl_3_-EDTA with agarose for (a) 0.0065 T, (b) 0.0475 T, (c) 0.05 T, (d) 0.064 T, (e) 0.55 T. Target tissue $T1$ and $T2$ times (stars; Blood=red, CSF=maroon, Fat=blue, GM=black, WM=gray) are shown. Each mimic measurement is shown with the same color as its tissue, and twice the standard deviation is plotted as error bars. Mixing models are displayed via constant agarose concentration lines (gray) and constant GdCl_3_-EDTA concentration lines (blue-green). Dashed gray line represents $T1=T2$. 26](#_Toc159491061)

[**Figure S16** $T1$ and $T2$ mimic sample measurements and mixing models for MnCl_2_ with agarose for (a) 0.0065 T, (b) 0.0475 T, (c) 0.05 T, (d) 0.064 T, (e) 0.55 T. Target tissue $T1$ and $T2$ times (stars; Blood=red, CSF=maroon, Fat=blue, GM=black, WM=gray) are shown. Each mimic measurement is shown with the same color as its tissue, and twice the standard deviation is plotted as error bars. Mixing models are displayed via constant agarose concentration lines (gray) and constant MnCl_2_ concentration lines (blue-green). Dashed gray line represents $T1=T2$. 27](#_Toc159491062)

# Table Captions

[**Table S1** Test samples (52 total) along with the paramagnetic salt contained in each sample that were used for fitting the mixing models. Samples that required heating and had evaporative loss correction are indicated with a Y, while samples that did not have evaporative loss correction are indicated with N. Four samples (indicated by [N, Y] in the evaporative correction column) were made twice: once with evaporative loss correction, once without. 6](#_Toc159491063)

[**Table S2** Relevant scan parameters for $T1$ and $T2$ measurements, for each fixed field strength and system. When multiple parameters are given, the chosen parameters were dependent on the expected $T1$ and $T2$ times of the sample being measured. 8](#_Toc159491064)

[**Table S3** Model fitting initialization values and limits for each fit parameter ***** To achieve a fit for CSF and GM, $Ci$ was allowed to vary down to 1x10^6^ s^-2^. ****** To achieve a fit for GM, B was initialized to 10 s^-1^. 14](#_Toc159491065)

[**Table S4** Fitting results for tissue dispersion models. ***** For blood and GM, this was allowed to range down to 1x10^6^. 15](#_Toc159491066)

[**Table S5** Target tissue T1 and T2 times for CuSO_4_ and agarose tissue mimics, along with CuSO_4_ and agarose concentrations. Measurements for T1 and T2 for each mimic sample at each field strength are listed, as well as the error of the measurement from both the target relaxation times. 28](#_Toc159491067)

[**Table S6** Target tissue T1 and T2 times for GdCl_3_-EDTA and agarose tissue mimics, along with GdCl_3_-EDTA and agarose concentrations. Measurements for T1 and T2 for each mimic sample at each field strength are listed, as well as the error of the measurement from both the target relaxation times. 29](#_Toc159491068)

[**Table S7** Target tissue T1 and T2 times for MnCl_2_ and agarose tissue mimics, along with MnCl_2_ and agarose concentrations. Measurements for T1 and T2 for each mimic sample at each field strength are listed, as well as the error of the measurement from both the target relaxation times. 30](#_Toc159491069)

[**Table S8** Target tissue T1 and T2 times for NiCl_2_ and agarose tissue mimics, along with NiCl_2_ and agarose concentrations. Measurements for T1 and T2 for each mimic sample at each field strength are listed, as well as the error of the measurement from both the target relaxation times. 31](#_Toc159491070)

# Methods

## Test Samples Prepared

| **Sample Name** | **Evaporation Correction**  **[N, Y, NA]** | **Paramagnetic Salt** |
| --- | --- | --- |
| 0.1 mmol/L CuSO_4_ | NA | CuSO_4_ |
| 0.1 mmol/L CuSO_4_ in 0.25% agarose mass concentration (%) | N | CuSO_4_ |
| 0.1 mmol/L CuSO_4_ in 0.5% agarose mass concentration (%) | N | CuSO_4_ |
| 0.25 mmol/L CuSO_4_ | NA | CuSO_4_ |
| 0.25 mmol/L CuSO_4_ in 0.25% agarose mass concentration (%) | N | CuSO_4_ |
| 0.25 mmol/L CuSO_4_ in 0.5% agarose mass concentration (%) | N | CuSO_4_ |
| 0.5 mmol/L CuSO_4_ | NA | CuSO_4_ |
| 0.75 mmol/L CuSO_4_ in 0.1% agarose mass concentration (%) | N | CuSO_4_ |
| 0.75 mmol/L CuSO_4_ in 0.25% agarose mass concentration (%) | N | CuSO_4_ |
| 1 mmol/L CuSO_4_ | NA | CuSO_4_ |
| 1 mmol/L CuSO_4_ in 0.1% agarose mass concentration (%) | N | CuSO_4_ |
| 1 mmol/L CuSO_4_ in 0.25% agarose mass concentration (%) | N | CuSO_4_ |
| 1 mmol/L CuSO_4_ in 1% agarose mass concentration (%) | [N, Y] | CuSO_4_ |
| 1 mmol/L CuSO_4_ in 2% agarose mass concentration (%) | N | CuSO_4_ |
| 2 mmol/L CuSO_4_ | NA | CuSO_4_ |
| 0.025 mmol/L GdCl_3_-EDTA | NA | GdCl_3_-EDTA |
| 0.025 mmol/L GdCl_3_-EDTA in 0.75% agarose mass concentration (%) | Y | GdCl_3_-EDTA |
| 0.025 mmol/L GdCl_3_-EDTA in 1.5% agarose mass concentration (%) | N | GdCl_3_-EDTA |
| 0.05 mmol/L GdCl_3_-EDTA | NA | GdCl_3_-EDTA |
| 0.05 mmol/L GdCl_3_-EDTA in 0.75% agarose mass concentration (%) | Y | GdCl_3_-EDTA |
| 0.05 mmol/L GdCl_3_-EDTA in 1.5% agarose mass concentration (%) | N | GdCl_3_-EDTA |
| 0.1 mmol/L GdCl_3_-EDTA | NA | GdCl_3_-EDTA |
| 0.1 mmol/L GdCl_3_-EDTA in 0.75% agarose mass concentration (%) | Y | GdCl_3_-EDTA |
| 0.1 mmol/L GdCl_3_-EDTA in 1.5% agarose mass concentration (%) | N | GdCl_3_-EDTA |
| 0.14 mmol/L GdCl_3_-EDTA in 1.5% agarose mass concentration (%) | [N, Y] | GdCl_3_-EDTA |
| 0.6 mmol/L GdCl_3_-EDTA In 1.3% agarose mass concentration (%) | N | GdCl_3_-EDTA |
| 0.028 mmol/L MnCl_2_ in 0.75% agarose mass concentration (%) | Y | MnCl_2_ |
| 0.028 mmol/L MnCl_2_ in 1.5% agarose mass concentration (%) | Y | MnCl_2_ |
| 0.131 mmol/L MnCl_2_ in 1.25% agarose mass concentration (%) | Y | MnCl_2_ |
| 0.131 mmol/L MnCl_2_ in 1.5% agarose mass concentration (%) | Y | MnCl_2_ |
| 0.54 mmol/L MnCl_2_ in 0.75% agarose mass concentration (%) | Y | MnCl_2_ |
| 0.54 mmol/L MnCl_2_ in 1.25% agarose mass concentration (%) | Y | MnCl_2_ |
| 1.5 mmol/L NiCl_2_ in 0.1% agarose mass concentration (%) | N | NiCl_2_ |
| 1.5 mmol/L NiCl_2_ in 0.75% agarose mass concentration (%) | Y | NiCl_2_ |
| 1.5 mmol/L NiCl_2_ in 1.5% agarose mass concentration (%) | Y | NiCl_2_ |
| 1.5 mmol/L NiCl_2_ in 4% agarose mass concentration (%) | N | NiCl_2_ |
| 10 mmol/L NiCl_2_ | NA | NiCl_2_ |
| 2 mmol/L NiCl_2_ in 0.1% agarose mass concentration (%) | N | NiCl_2_ |
| 3 mmol/L NiCl_2_ in 0.1% agarose mass concentration (%) | N | NiCl_2_ |
| 4 mmol/L NiCl_2_ in 0.1% agarose mass concentration (%) | N | NiCl_2_ |
| 4 mmol/L NiCl_2_ in 0.75% agarose mass concentration (%) | Y | NiCl_2_ |
| 4 mmol/L NiCl_2_ in 1.2% agarose mass concentration (%) | [N, Y] | NiCl_2_ |
| 4 mmol/L NiCl_2_ in 1.5% agarose mass concentration (%) | Y | NiCl_2_ |
| 5 mmol/L NiCl_2_ | NA | NiCl_2_ |
| 8 mmol/L NiCl_2_ in 0.5% agarose mass concentration (%) | N | NiCl_2_ |
| 0.1% agarose mass concentration (%) | N |  |
| 0.5% agarose mass concentration (%) | N |  |
| 1% agarose mass concentration (%) | Y |  |
| 2% agarose mass concentration (%) | [N, Y] |  |
| 3.25% agarose mass concentration (%) | N |  |
| 4% agarose mass concentration (%) | N |  |
| Deionized water | NA |  |

**Table S1** Test samples (52 total) along with the paramagnetic salt contained in each sample that were used for fitting the mixing models. Samples that required heating and had evaporative loss correction are indicated with a Y, while samples that did not have evaporative loss correction are indicated with N. Four samples (indicated by [N, Y] in the evaporative correction column) were made twice: once with evaporative loss correction, once without.

## $\boldsymbol{T}_{\mathbf{1}}$ and $\boldsymbol{T}_{\mathbf{2}}$ Measurement

Quantitative imaging protocol details for each fixed-field measurement system used in this study are listed in **Table S2**. Timing parameters for each system were chosen with respect to hardware limitations and the expected relaxation times to be measured for that field. In general, for $T_{1}$ the repetition time or final delay (for NMR measurements) was chosen to be greater than 5*$T_{1}$ for the sample being tested. Additional details for the measurement protocols used for each system are listed below, as well as details for the FFC system.

| **Protocol** | **System** | **Sequence Type** | **Timing Parameters (s)** | **Repetition Time / Last Delay (s)** | **Image Res (mm)** | **Slice Thickness (mm)** |
| --- | --- | --- | --- | --- | --- | --- |
| T_1_ | 0.0065 T MRI | IR  spectroscopic | TI: 40 TIs starting at 0.001 s, linearly increasing with step size of 0.04 s, 0.06 s, 0.08 s, or 0.120 s. | 2, 5, or 10 | NA | NA |
|  | 0.0475 T MRI | IR spectroscopic | TI: 0.0285 s to 0.2085 s in 0.020 s increments, and 0.3085 s to 2.8085 s in 0.1 s increments | 5 | NA | NA |
|  | 0.05 T MRI | IR  spectroscopic | TI: 20 logarithmically increasing TIs from 0.005 s to 20 s | 20 | NA | NA |
|  | 0.064 T MRI | IR  3D | TI: 0.1, 0.2, 0.3, 0.4, 0.5, 0.6, 0.7, 0.8, 0.9, 1.1, 1.3, 1.5, 1.8, 2.1, 2.5 | 3 | 1.6 x 1.6 | 5 |
|  | 0.55 T MRI | IR  2D slice | TI: 0.05, 0.1, 0.2, 0.5, 0.9, 1.8, 3, 6 or TI: 0.05, 0.1, 0.2, 0.3, 0.5, 0.7, 0.9, 1.2, 1.5, 1.8, 2.2, 2.4, 3, 6 or TI: 0.05, 0.1, 0.2, 0.3, 0.5, 0.7, 0.9, 1.2, 1.5, 1.8, 2.2, 2.4, 3 | 10 | 0.86 x 0.856 | 10 |
|  | 0.0065 T, 0.064 T, 0.55 T NMR | IR  spectroscopic | TI: 20 exponentially increasing Tis in steps of [0.001*x to x] with x ranging from 1 s to 15 s depending on expected T_1_ | 5*T_1expected_ | NA | NA |
| T_2_ | 0.0065 T MRI | CPMG  spectroscopic | TE: 50 linearly increasing TEs with step size one of 0.015 s, 0.01 s, or 0.009 s | 2 | NA | NA |
|  | 0.0475 T MRI | CPMG spectroscopic | TE: linearly increasing with step size of 0.0075 s or 0.014 s | 5 | NA | NA |
|  | 0.05 T MRI | CPMG  spectroscopic | TE: 20 logarithmically increasing TEs from 0.005 s to 10 s | 20 | NA | NA |
|  | 0.064 T MRI | SE  3D | TE: 0.037, 0.111, 0.185, 0.259, 0.333, 0.407, 0.480, 0.554, 0.628, 0.702 | 3 | 1.5 x 1.5 | 5 |
|  | 0.55 T  MRI | SE  2D slice | TE: 0.01, 0.025, 0.05, 0.075, 0.1, 0.2, 0.3, 0.5, 0.7, 0.9, 1.0 or TE: 0.01, 0.025, 0.05, 0.1, 0.2, 0.5, 1 | 10 | 0.86 x 0.856 | 10 |
|  | 0.0065 T, 0.064 T, 0.55 T NMR | CPMG  spectroscopic | TE: 20 linearly increasing steps of [0.05*x to x] with x ranging from 0.04 s to 3.76 s depending on expected T_2_ | 5*T_1expected_ | NA | NA |

**Table S2** Relevant scan parameters for $T_{1}$ and $T_{2}$ measurements, for each fixed field strength and system. When multiple parameters are given, the chosen parameters were dependent on the expected $T_{1}$ and $T_{2}$ times of the sample being measured.

$T_{1}$ and $T_{2}$ were fit by each site, generally using standard signal models for inversion-recovery (IR) and spin-echo (SE) sequences. Unless noted otherwise, the signal models to fit $T_{1}$ and $T_{2}$ were:

$S_{i}=S_{0}\left( 1-2e^{- \frac{{TI}_{i}}{T_{1}}} \right)$ [S.1]

$S_{i}=S_{0}e^{- \frac{TE_{i}}{T_{2}}}$ [S.2]

with $S_{i}$ the measured signal for the $i$th $TI$ or $TE$, $S_{0}$ the nominal signal, and $T_{1}$ and $T_{2}$ the values to fit.

### NMR System: 0.0065 T – Additional Details

Measurements were made using an ultra-low field scanner with an absolute homogeneity of better than 0.25 Hz (0.9 ppm) over a 10 mm NMR sample tube.

### MRI System: 0.0475 T – Additional Details

Measurements were made using a Tecmag Redstone spectrometer (TNMR Software version 3.4.31). All samples were at room temperature of approximately 20 °C during measurement. All measurements were performed with a uniform solenoid, which fully contained the 50 ml sample. Single voxel measurements were taken for both $T_{1}$ and $T_{2}$.

$S_{0}$ and either $T_{1}$ or $T_{2}$ were simultaneously fit using the MATLAB (The MathWorks Inc., Natick, MA, USA) function fitnlm.

### MRI System: 0.064 T – Additional Details

$T_{1}$ maps were acquired using a research version of the Hyperfine $T_{1}$-weighted IR 3D fast spin echo (FSE) sequence. Images were acquired with a field of view of 22 cm x 18 cm x 18 cm. Each IR scan time was 11 minutes and 13 seconds, leading to a total $T_{1}$ mapping scan time of 168 minutes and 15 seconds. $T_{2}$ maps were acquired using a research version of a Hyperfine $T_{2}$-weighted 3D FSE sequence. Images were acquired with a field of view of 22 cm x 18 cm x 18 cm. The $T_{2}$ mapping sequence scan time was 17 minutes and 8 seconds.

Measurements were made using a custom Hyperfine-provided phantom that held 50 ml sample tubes. Regions of interest (ROIs) were selected using an automated protocol that searched for circles of the expected 50 ml tube size in each image slice. Once each tube was identified for each slice, the ROI of each tube was limited to a central circular region of radius equal to half the tube’s image radius. $T_{1}$ was fit using data from all voxels located in each ROI simultaneously, which resulted in one $T_{1}$ per ROI and per slice. $T_{2}$ values came directly from the Hyperfine software, and the mean of the $T_{2}$ value for all voxels in each ROI was calculated to give one $T_{2}$ value per ROI per slice. Finally, the six central slices of the phantom were selected due to their clean ROI tube segmentations compared to slices near the ends of the tubes, which had ROI segmentations that sometimes included the ends of the tube. The final reported $T_{1}$ and $T_{2}$ values represent the mean and standard deviation of each tube’s ROI over these central six slices.

$T_{1}$ was calculated for each voxel using the lmfit package in Python for the IR model^1^:

$S_{i}=S_{0}\left| 1-\left( 1+d \right)e^{- \frac{{TI}_{i}}{T_{1}}}+e^{- \frac{TR}{T_{1}}} \right|$ [S.3]

where $T_{1}$ is the target value for the fit and $d$ is a scale factor to account for imperfect inversion. The $T_{2}$ map was calculated using a Hyperfine protocol that used SciPy optimize curve_fit in Python.

### MRI System: 0.55 T – Additional Details

ROIs were manually selected by drawing a conservative circle on the central part of one tube and manually shifting the ROI to other samples. This ensures that each ROI has roughly the same number of pixels. The same ROIs were used for both $T_{1}$ and $T_{2}$.

$T_{1}$ was calculated using the three-parameter model:

$S = S_{0} * (1 - Be^{- TI/T_{1}})$ [S.4]

and $T_{2}$ map was calculated using the three-parameter model:

$S_{i}=S_{0}e^{- \frac{TE}{T_{2}}}+C$ [S.5]

where $B$ and $C$ are parameters that account for imperfect inversion and noise in the signal.

### NMR Systems: 0.0065 T, 0.064 T, 0.55 T – Additional Details

NMR measurements were made using a Redstone spectrometer and TNMR software (Tecmag, Houston, TX, USA). A custom RF copper solenoid coil was used for each system, which were designed to allow perfluorocarbon coolant (TMC Industries, Inc., Waconia, MN, USA PN: FC-40) to flow around the sample for temperature control. For the 0.064 T NMR system, the samples for the NMR measurements were kept at 21.3 °C, the approximate temperature of the laboratory housing the 0.064 T MRI system. For the 0.0065 T and 0.55 T NMR systems, the samples for the NMR measurements were kept between 20.0 °C and 26.0 °C, to cover a temperature range seen in most laboratories.

Three repeated measurements were acquired for both $T_{1}$ and $T_{2}$, and the reported mean and standard deviation for each sample were calculated over the three replicate measurements. The NMR measurements used $TR$ > 5*$T_{1}$, and thus $T_{1}$ was calculated using a simplified form of Eq. S.3:

$S_{i}=S_{0}\left| 1-\left( 1+d \right)e^{- \frac{{TI}_{i}}{T_{1}}} \right|.$ [S.6]

### FFC $T_{1}$ Measurement: 0.0007 T to 0.55 T – Additional Details

^1^H longitudinal relaxation time $T_{1}$ was measured from 0.0007 T to 0.55 T using a Spinmaster FFC2000 1T Relaxometer (Stelar s.r.l., Mede (PV), Italy). $T_{1}$ was determined using the standard prepolarized (PP) and nonpolarized (NP) sequences^2,3^ and using the following equation:

$S\left( \tau\right)= S_{o}\left( B_{r} \right)+\left[ S_{o}\left( B_{p} \right)- S_{o}\left( B_{r} \right) \right]e^{\frac{-\tau}{T_{1}}}.$ [S.7]

Here S(τ) is the signal detected after the relaxation interval time τ, S_o_(B_r_) and S_o_(B_p_) are the signals corresponding to the equilibrium magnetization at the relaxation (B_r_) and polarization (B_p_) fields, respectively. The polarization and acquisition magnetic fields kept constant values of 0.35 T and 0.38 T respectively, for all experiments. The field slew rate was 0.31 T/ms and the switching time was 3 ms. $T_{1}$ values were measured at 23^o^C for the phantom samples and at 37^o^C for GM and WM samples. Experimental uncertainties were typically less than 2%, which lie within the size of the data points in graphs.

## Proof of Concept: Tissue Mimics using Mixing Model Recipes

### Tissue Relaxation Data

Tissue relaxation times were compiled into a database to aid in the modeling of relaxation dispersion. Data were compiled from sources found through searching the PubMed and Google Scholar databases for tissue relaxation measurements below 1 T, but included studies up to 7 T. The measurements span 40 years for fields between 1.71 μT to 21.1 T and include 32 tissue types and 183 unique sample compositions. When provided, the parameters recorded in the database included: tissue or sample composition, the quantitative measurement value and quantitative variable (e.g., $T_{1}$ (s), $T_{2}$ (s)), field strength (T), temperature (K) of the sample, and relevant sequence parameters. When data were not explicitly given in numerical form, the WebPlotDigitizer^4^ package was used to extract data from plots. Forty-five references from the database were used for tissue relaxation modeling.^5–49^ For blood, most references measured sagittal sinus blood^20,21,49^. One measured arterial blood^38^, two were *ex vivo* studies^19,41^, and one was a review^37^.

To supplement literature measurements, $T_{1}$ measurements were acquired for *ex vivo* WM and GM samples. *Ex vivo* samples were prepared in accordance with Institutional Review Board guidelines. Three GM samples and two WM samples were excised (see **Figure S1** for sample locations). Prior to excision, brain tissue was warmed over 24 hours from -80°C to -20°C, and subsequently over another 24 hours to 4°C. To preserve *in vivo* relaxation properties, the number of freeze/thaw cycles was kept less than 3, and samples were not fixed. Samples were stored at -80°C in NMR tubes, filled with the sample to approximately 25.4 mm height, with the remainder of the tube filled with Teflon (DuPont, Wilmington, DE, USA) to minimize evaporation. Tubes were capped and sealed with parafilm prior to storage. Samples were shipped on dry ice to the measurement site and measured upon receipt.


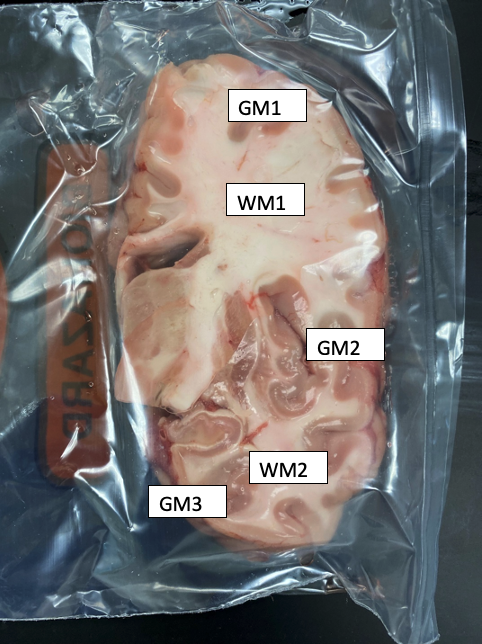


**Figure S1** Photograph of excised brain locations. Three samples of GM were excised, and two samples of WM were excised.

### Dispersion Models

$T_{1}$ and $T_{2}$ dispersion models were fit using Python’s lmfit package for each tissue using the tissue relaxation data described above.

Many $T_{1}$ dispersion models have been described.^36,50–52^ Here, a function comprised of up to three modified Lorentzian functions was used, each with an adjustable exponential parameter to account for dispersion broadening,^20,53^ and each representing a component of tissue that can each be thought of as a fast, intermediate, or slow dynamical process corresponding to free, bound, or tightly bound water.^31,34,54^

$R_{1}=\frac{1}{T_{1}}=A+\sum_{i=1}^{3} C_{i}\left( \frac{\tau_{i}}{\left( 1+\left( \omega\tau_{i} \right) \right)^{\beta_{i}}}+\frac{{4\tau}_{i}}{\left( 1+\left( 2\omega\tau_{i} \right) \right)^{\beta_{i}}} \right)$ [S.8]

where $C_{i}$ (s^-2^) is the strength of the $i$^th^ component’s dipole-dipole coupling, $\tau_{i}$ (s) is the $i$^th^ component’s correlation time, $\omega$ (MHz) is the proton Larmor frequency, the $\beta_{i}$ parameter accounts for the $i$^th^ component’s dispersion broadening, $A$ (s^-1^) describes relaxation for processes on timescales less than 10^-9^ s,^53,54^ and $T_{1}$ (s) is the relaxation time. For some tissues with simple structures (e.g., CSF), three components may not be necessary but were modeled, sometimes yielding parameters with values close to zero.

$T_{2}$ was similarly fit using a sum of modified Lorentzian functions.^36,37,51^ The $T_{2}$ dispersion model for each tissue was modified from references^25,36^ to have the same number of components as $T_{1}$:

$R_{2}=\frac{1}{T_{2}}=B+\sum_{i=1}^{3} C_{i}\left( 1.5\tau_{i}+\frac{2.5\tau_{i}}{\left( 1+\left( \omega\tau_{i} \right) \right)^{\beta_{i}}}+\frac{\tau_{i}}{\left( 1+\left( 2\omega\tau_{i} \right) \right)^{\beta_{i}}} \right)$ [S.9]

with parameters defined as in **Equation S8**, with $B$ (s^-1^) describing $T_{2}$ relaxation for processes on timescales less than 10^-9^ s, and $T_{2}$ (s) as the relaxation time. Although $T_{2}$ is often thought to be field invariant due to the magnitude of the $J(0)$ term in most formulations of the spectral density^55^, at lower fields this term decreases and the other terms have greater contribution, resulting in $T_{2}$ dispersion. $T_{2}$ was modeled as a monoexponential decay.

The $T_{1}$ and $T_{2}$ models were fit simultaneously by minimizing the error in both models using global parametric optimization. For CSF, the data from two references^37,42^ were excluded from the model fitting based on inadequate experimental repetition times, which can bias the fit. For three literature data points (one each for fat, WM, and GM) that reported non-monoexponential decay, the largest fractional component was used. Due to a data entry error, a smaller fractional component was used for WM and GM. This data entry error affected the WM $T_{2}$ targets by 1.2% for all field strengths except for 0.55 T, which had a 1.7% difference from what the $T_{2}$ target would have been without the data entry error. For GM, the data entry affected the $T_{2}$ targets by 1.7%, 1.1%, 1.2%, 1.7%, and 4.1% for 0.0065 T, 0.0475 T, 0.05 T, 0.064 T, and 0.55 T, respectively.

**Table S3** lists the model parameters and their initialization values for each tissue, as well as the parameter limits of the fitting algorithm. While $\tau_{i}$ can vary up to the length of seconds for some chemical materials, human tissue $\tau_{i}$ tends to vary between 1x10^-2^ s to 1x10^-8^ s.^52^ Studies have measured water correlation time in the picosecond range;^56^ therefore, $\tau_{i}$ was allowed to vary down to 1x10^-13^ s. $C_{i}$ has been typically found to be between 1x10^7^ s^-2^ and 1x10^10^ s^-2^;^57^ however, to achieve fits for GM and blood, $C_{i}$ was allowed to vary down to 1x10^6^ s^-2^ for those tissues. The parameter $\beta_{i}$ is a dispersion broadening parameter and should vary between 0 and 2.^53^ A and B were allowed to vary between 1x10^-10^ s^-1^ and 10 s^-1^. Fits were achieved with A initialized to 0.5 s^-1^ and B initialized to 1 s^-1^ for all tissues except for GM, which could not achieve a fit unless B was initialized to 10 s^-1^.

| **Model Definition** | **Parameter** | | | | |
| --- | --- | --- | --- | --- | --- |
|  | $\boldsymbol{C}_{\boldsymbol{i}}$ **(s^-2^)** | $\boldsymbol{\tau}_{\boldsymbol{i}}$ **(s)** | $\boldsymbol{\beta}_{\boldsymbol{i}}$ | $\boldsymbol{A}$ **(s^-1^)** | $\boldsymbol{B}$ **(s^-1^)** |
| Fit limits | *1x10^7^** *to 1x10^10^* | *1x10^-13^ to 1x10^-2^* | *0 to 2* | *1x10^-10^ to 10* | *1x10^-10^ to 10* |
| Initial conditions | [1x10^8^, 1x10^8^, 1x10^8^] | [1x10^-7^, 1x10^-8^, 1x10^-10^] | [1, 1, 1] | 0.5 | 1** |

**Table S3** Model fitting initialization values and limits for each fit parameter
***** To achieve a fit for CSF and GM, $C_{i}$ was allowed to vary down to 1x10^6^ s^-2^.
****** To achieve a fit for GM, B was initialized to 10 s^-1^.

**Table S4** lists the parameters for the fitted dispersion models of blood, CSF, fat, GM, and WM. For all tissues, A was smaller than B, indicating that the constant term for $R_{1}$ is shorter than for $R_{2}$ ($T_{1}\geq T_{2}$). The CSF model had overall shortest $\tau_{i}$, as expected. Similarly, blood had relatively short $\tau$ for two components (one of which had $\beta$ approximately zero), and a longer $\tau$ for one component, which could be representative of proteins. Fat had the longest $\tau_{i}$, as expected given its more rigid structure. Both WM and blood had a component with $\beta$ very close to zero. WM and blood had two $\tau_{i}$ that indicate a liquid-like and a structured component; however, blood’s $\beta$ values were much higher than WM’s $\beta$ values, indicating more dispersion broadening. This is expected due to WM’s complex nature. GM had the widest range of $\tau_{i}$, which may be representative of its complex cellular structure.

| **Tissue** | **Parameter** | | | | |
| --- | --- | --- | --- | --- | --- |
|  | $\boldsymbol{C}_{\boldsymbol{i}}$ **(s^-2^)**  *1x10^7^* to 1x10^10^* | $\boldsymbol{\tau}_{\boldsymbol{i}}$ **(s)**  *1x10^-13^ to 1x10^-2^* | $\boldsymbol{\beta}_{\boldsymbol{i}}$  *0 to 2* | $\boldsymbol{A}$ **(s^-1^)**  *1x10^-10^ to 10* | $\boldsymbol{B}$ **(s^-1^)**  *1x10^-10^ to 10* |
| Blood | [4.54x10^6^,  1.02x10^8^,  7.50x10^7^] | [1.30x10^-7^,  9.26x10^-10^,  6.60x10^-11^] | [1.04,  2.00,  8.61x10^-7^] | 1.98x10^-5^ | 2.79 |
| CSF | [1.00x10^7^,  1.00x10^7^,  1.00x10^7^] | [9.48x10^-9^,  1.00x10^-12^,  1.00x10^-12^] | [0.16,  1.14,  1.14] | 1.00x10^-10^ | 0.29 |
| Fat | [1.00x10^7^,  1.00x10^7^, 1.00x10^7^] | [1.74x10^-7^,  9.93x10^-8^,  5.51x10^-11^] | [0.13,  0.90,  0.047] | 4.91x10^-10^ | 3.87 |
| GM | [1.00x10^6^, 2.15x10^6^, 8.99x10^6^] | [1.91x10^-6^,  1.15x10^-12^,  1.00x10^-12^] | [0.41,  1.99,  1.31] | 0.099 | 9.01 |
| WM | [1.00 x10^7^, 1.60 x10^8^,  1.00 x10^7^] | [1.62x10^-7^,  1.70x10^-10^,  7.61x10^-11^] | [0.52,  2.64x10^-14^,  0.14] | 2.67x10^-4^ | 8.52 |

**Table S4** Fitting results for tissue dispersion models. ***** For blood and GM, this was allowed to range down to 1x10^6^.

To further examine the dispersion models, the fits and data for the fits are shown in **Figure 1**. $T_{1}$ measurements for blood and CSF were difficult to find in literature. For $T_{2}$, there was a dearth of measurements for all tissues. To improve the dispersion models, all models were fit using all data in the database, which sometimes extended above 0.55 T. This was especially important for blood $T_{1}$, CSF $T_{1}$, and many of the tissues’ $T_{2}$ models, due to limited relaxation data available below 0.55 T. The *ex vivo* $T_{1}$ measurements for WM and GM followed a similar trend to literature data and at times were outside of ±10% of the models. The $T_{1}$ dispersion models spanned a wider range of values than the range of values from the $T_{2}$ models.

Challenges were encountered when compiling literature relaxation measurements and fitting dispersion models. Past low field quantitative measurements have suffered from hardware limitations, resulting in trade-offs between feasibility and accuracy.^37,42^ Variations in demographics and regions of interest can cause variations in literature measurements, independent of field. Finally, there are very few $T_{2}$ measurements at low field. Thus, in this study many $T_{2}$ measurements from literature were outside of ±10% of the dispersion model, and the fitted dispersion models therefore do not fit the data closely. Adding ex vivo $T_{2}$ measurements could help improve the models. In general, the community at large should address the need for more $T_{2}$ studies of biological tissues at low field.


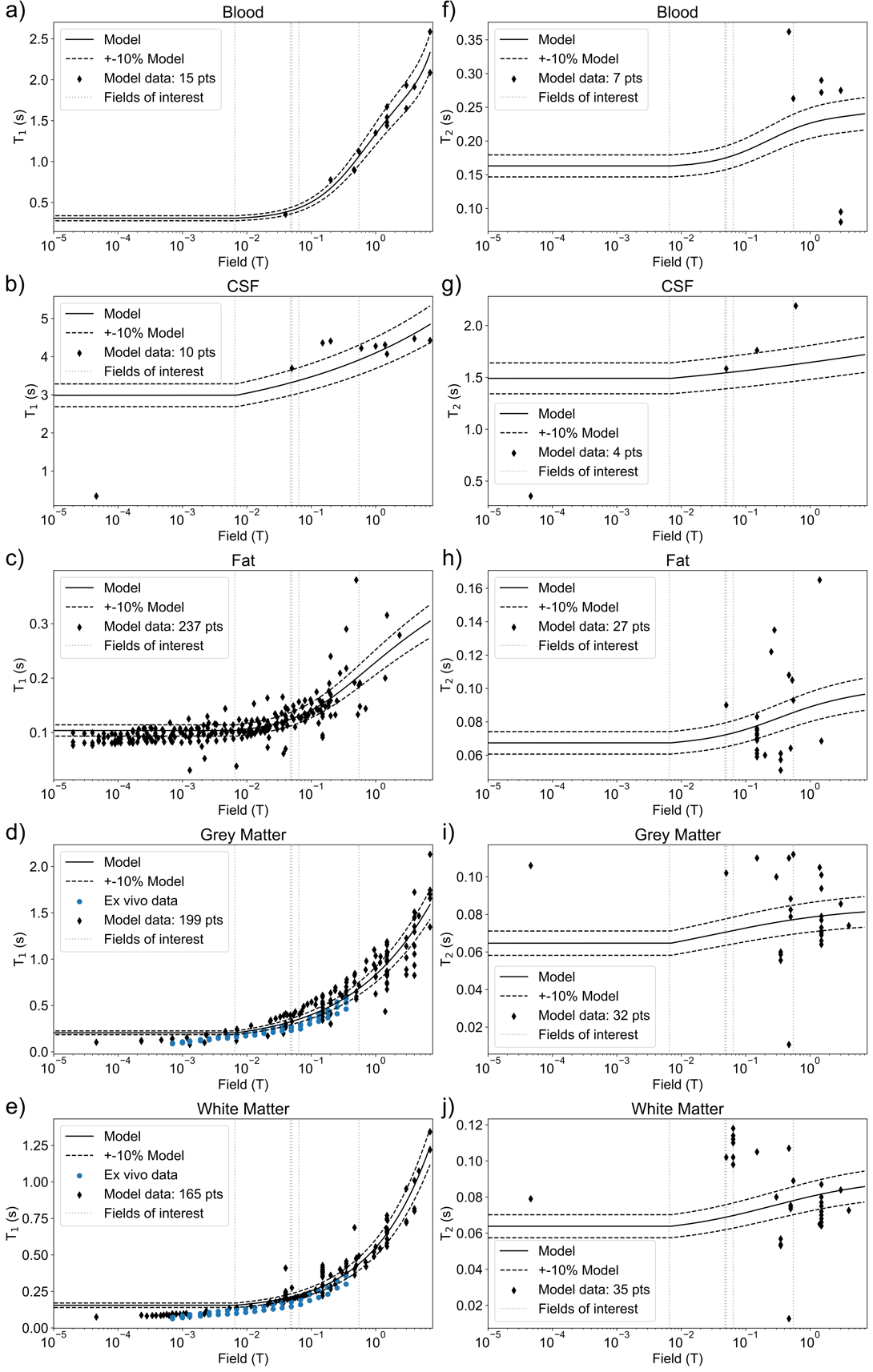


**Figure S2** (a-e) $T_{1}$ and (f-j) $T_{2}$ dispersion models for (a,f) blood, (b,g) CSF, (c,h) fat, (d,i) GM, and (e,j) WM are shown in the solid line. A 10% variation of the model is shown in dashed lines, and the data used to create the fit is displayed in blue circles for the ex vivo data collected in this study and in black diamonds for the literature data. The number of literature data points are indicated in each legend. The fields of interest for this study (0.0065 T, 0.0475 T, 0.05 T, 0.064 T, 0.55 T) are indicated by vertical dotted grey lines.

# Results

## $\boldsymbol{T}_{\mathbf{1}}$ and $\boldsymbol{T}_{\mathbf{2}}$ Mixing Models


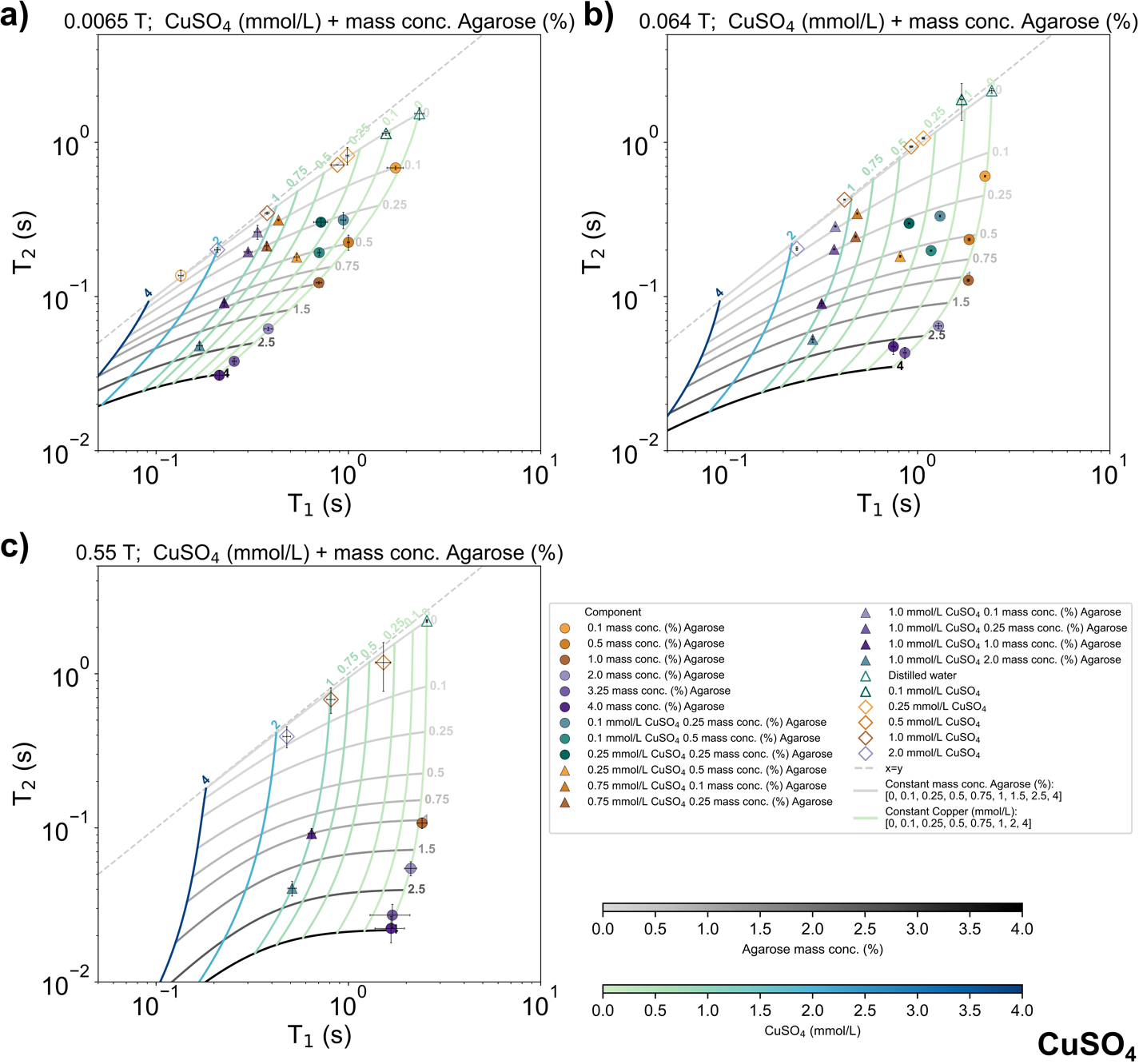


**Figure S3** $T_{1}$ and $T_{2}$ test sample measurements and mixing models for CuSO_4_ + agarose for (a) 0.0065 T, (b) 0.064 T, (c) 0.55 T. Mixing models are displayed via constant agarose concentration lines (solid) and constant CuSO_4_ concentration lines (dashed). Dashed gray line represents T1=T2, and twice the standard deviation is plotted as error bars.


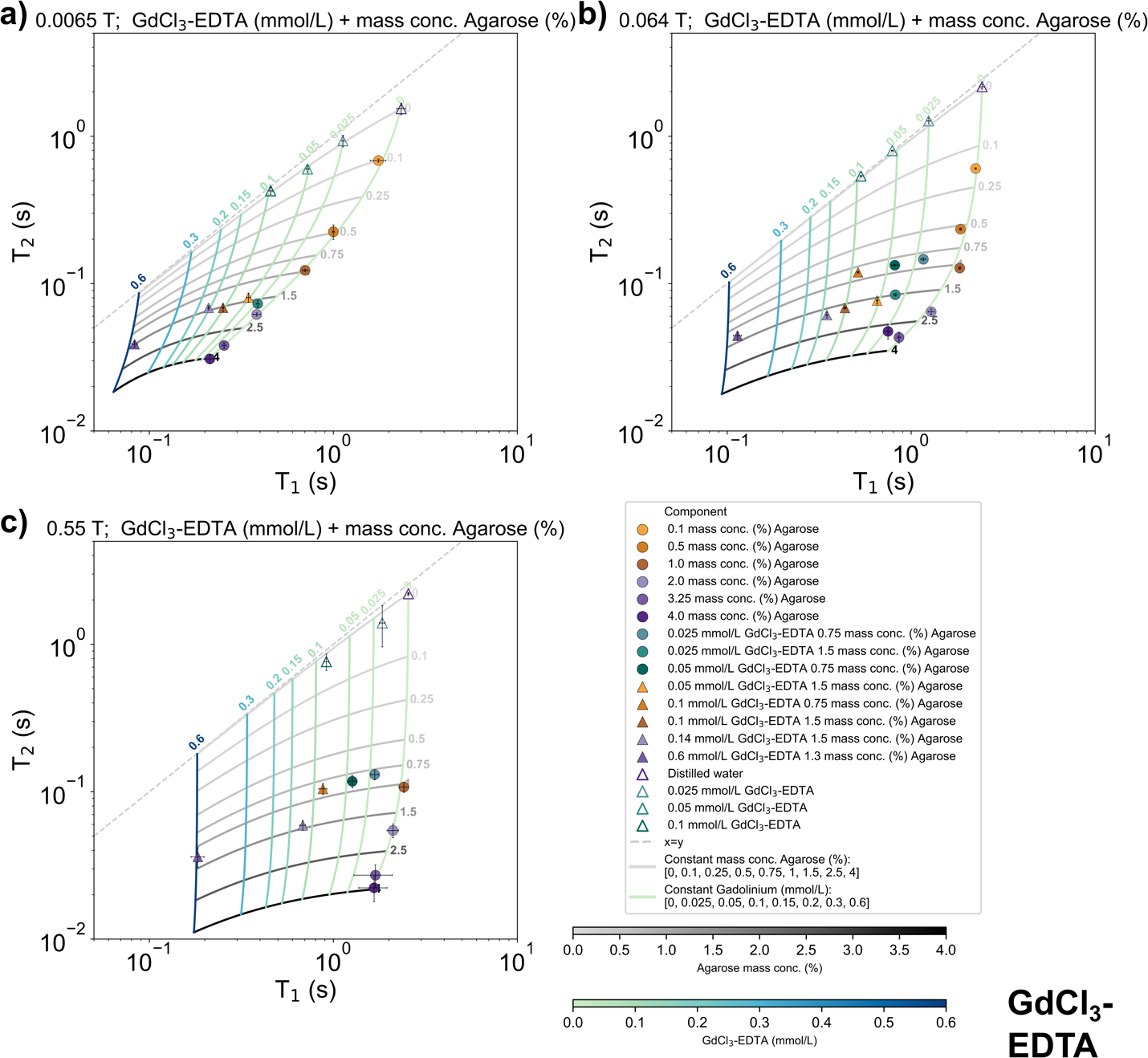


**Figure S4** $T_{1}$ and $T_{2}$ test sample measurements and mixing models for GdCl_3_-EDTA + agarose for (a) 0.0065 T, (b) 0.064 T, (c) 0.55 T. Mixing models are displayed via constant agarose concentration lines (solid) and constant GdCl_3_-EDTA concentration lines (dashed). Dashed gray line represents T1=T2, and twice the standard deviation is plotted as error bars.


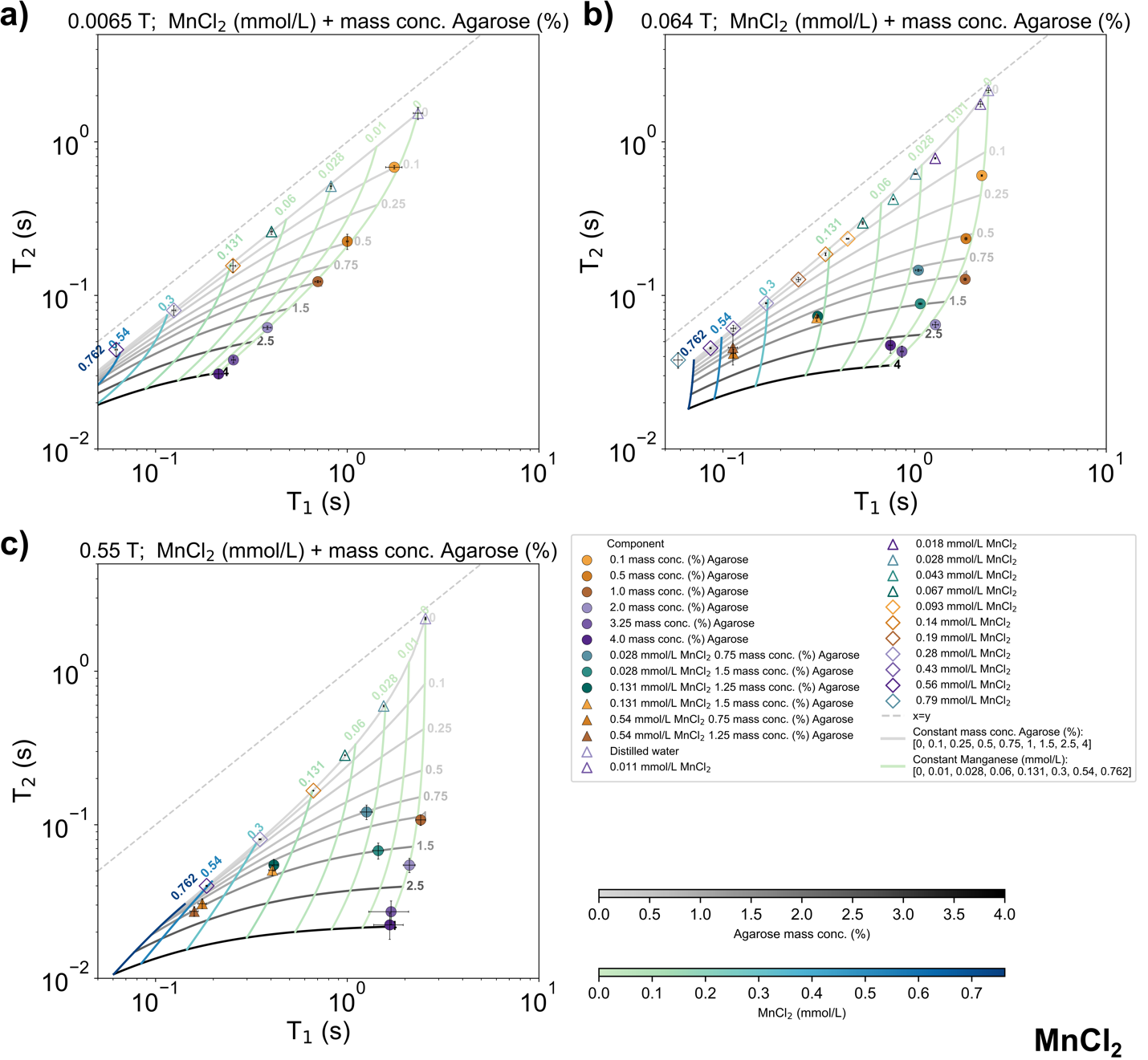


**Figure S5** $T_{1}$ and $T_{2}$ test sample measurements and mixing models for MnCl_2_ + agarose for (a) 0.0065 T, (b) 0.064 T, (c) 0.55 T. Mixing models are displayed via constant agarose concentration lines (solid) and constant MnCl_2_ concentration lines (dashed). Dashed gray line represents T1=T2, and twice the standard deviation is plotted as error bars.


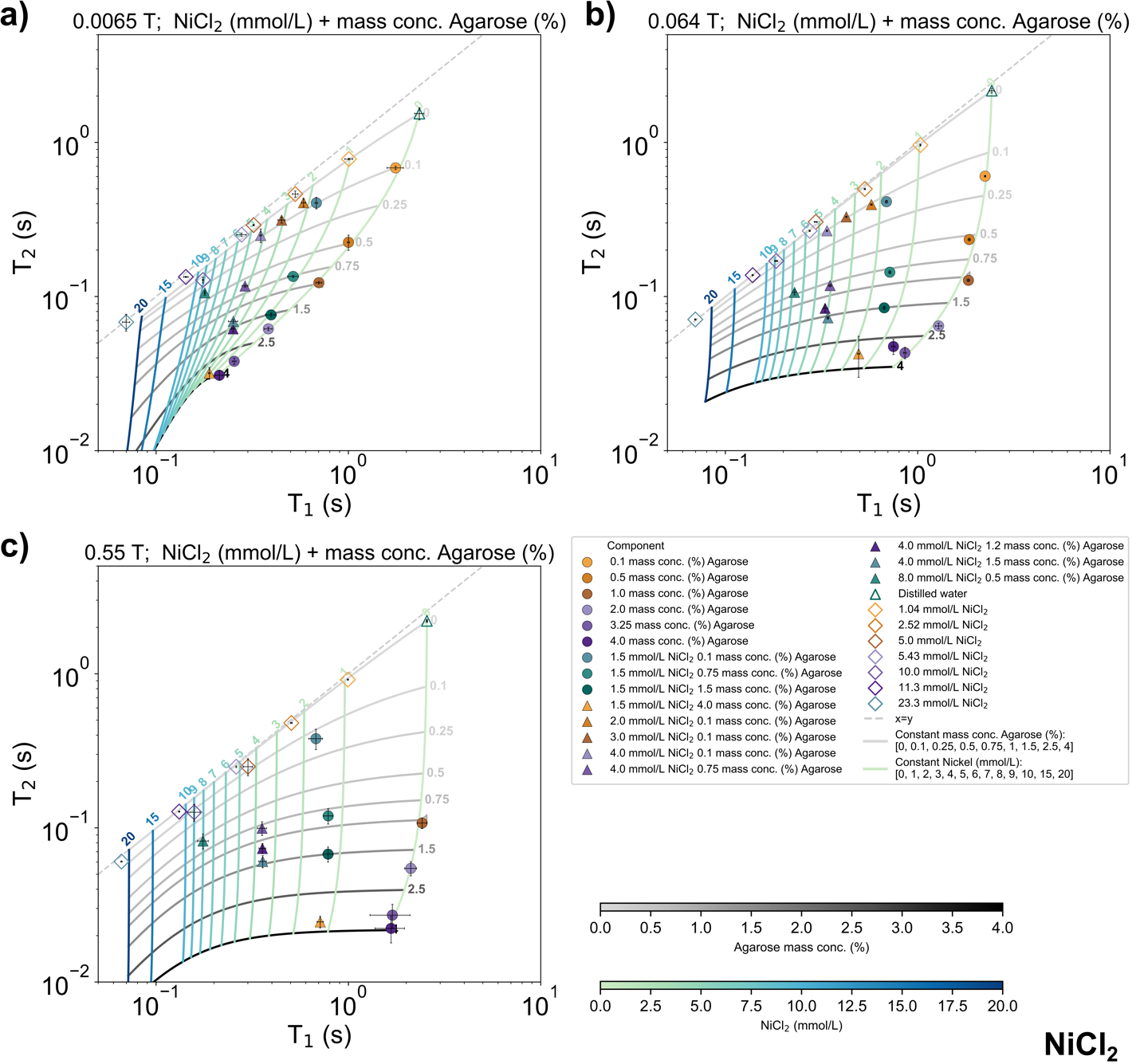


**Figure S6** $T_{1}$ and $T_{2}$ test sample measurements and mixing models for NiCl_2_ + agarose for (a) 0.0065 T, (b) 0.064 T, (c) 0.55 T. Mixing models are displayed via constant agarose concentration lines (solid) and constant NiCl_2_ concentration lines (dashed). Dashed gray line represents T1=T2, and twice the standard deviation is plotted as error bars.

## $\boldsymbol{T}_{\mathbf{1}}$ and $\boldsymbol{T}_{\mathbf{2}}$ Measurement Evaluation Metrics

### Variation in Repeatability


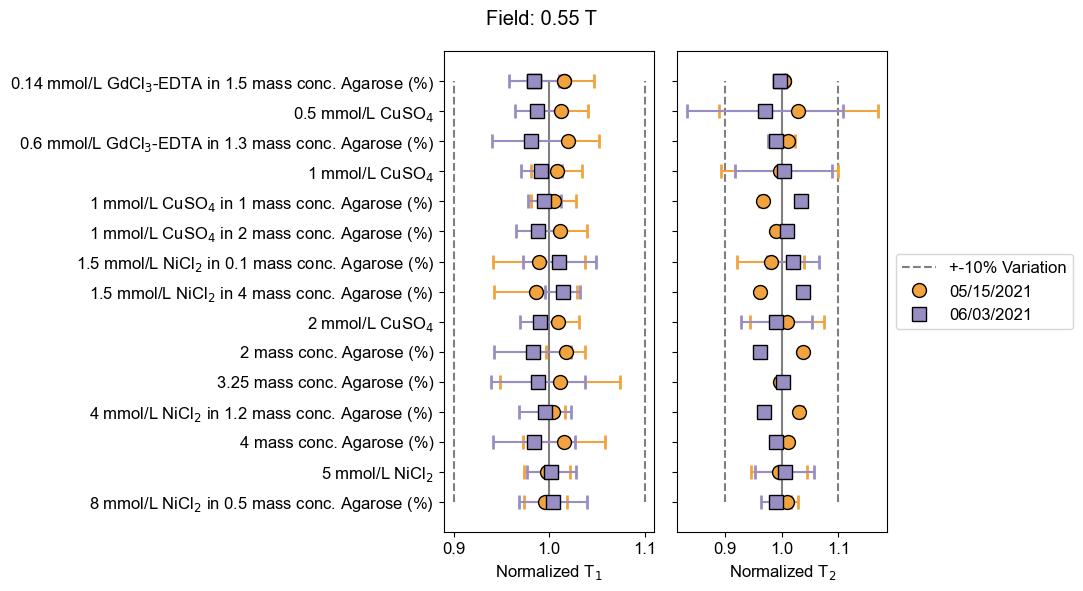


**Figure S7** Normalized $T_{1}$ (left) and $T_{2}$ (right) variation between repeat measurements for 15 samples at 0.55 T. Data are normalized to the average of the mean values for each sample. The coefficient of variation is plotted as error bars. The date of measurements is indicated by the marker.

### Variation in Protocol-Dependence


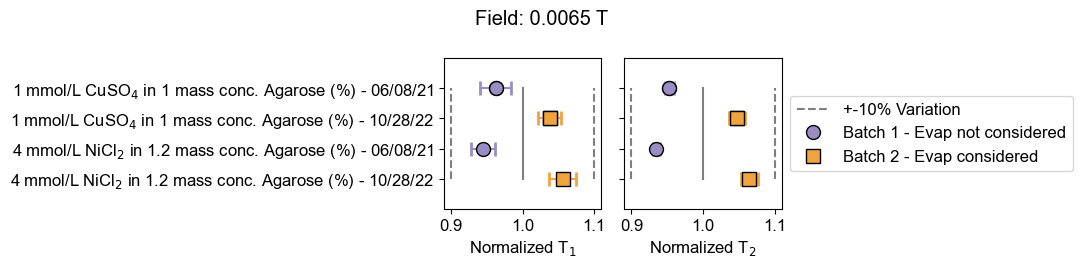


**Figure S8** Normalized $T_{1}$ (left) and $T_{2}$ (right) variation between protocols for two samples at 0.0065 T. Data are normalized to the average of the mean values for each sample, for each protocol. The coefficient of variation is plotted as error bars. The date of measurements is indicated in the label. Each of these samples had an agarose component, and batch 1 did not consider evaporative water losses in the synthesis protocol, whereas batch 2 did consider evaporative water loss.


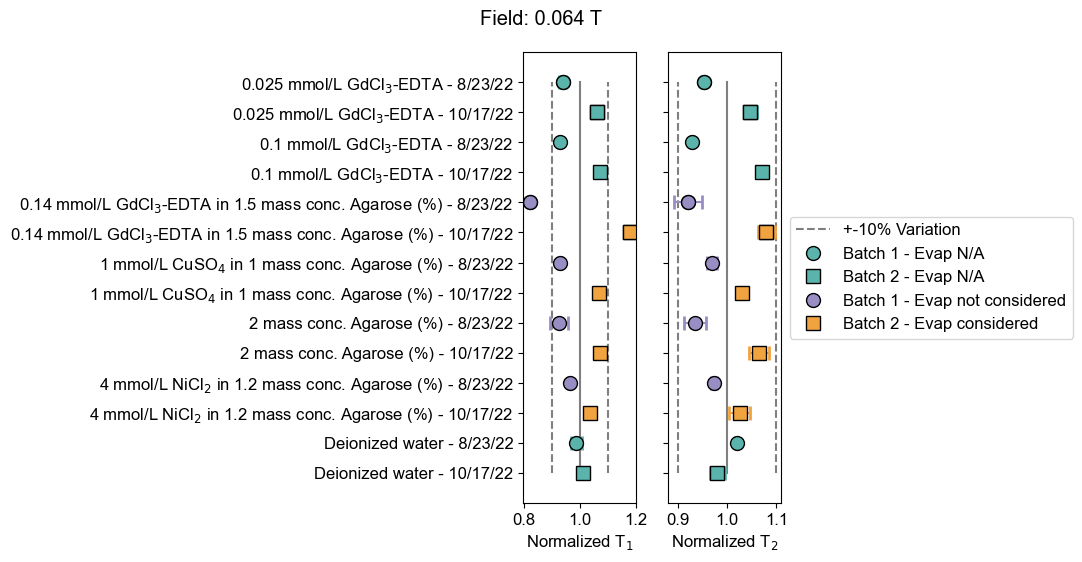


**Figure S9** Normalized $T_{1}$ (left) and $T_{2}$ (right) variation between protocols for 7 samples at 0.064 T. Data are normalized to the average of the mean values for each sample, for each protocol. The coefficient of variation is plotted as error bars. The date of measurements is indicated in the label. Four samples had an agarose component, and batch 1 did not consider evaporative water losses in the synthesis protocol, whereas batch 2 did consider evaporative water loss. Three samples had no agarose component and evaporative water loss was not applicable for the synthesis protocol.


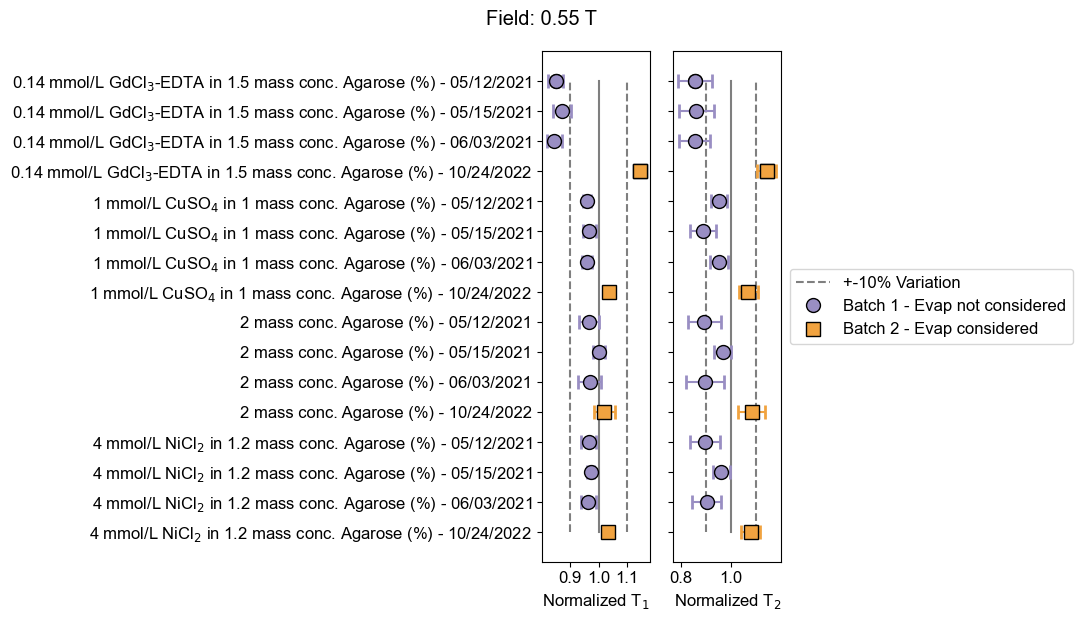


**Figure S10** Normalized $T_{1}$ (left) and $T_{2}$ (right) variation between protocols for 4 samples at 0.55 T. Data are normalized to the average of the mean values for each sample, for each protocol. The coefficient of variation is plotted as error bars. The date of measurements is indicated in the label. All samples had an agarose component, and batch 1 did not consider evaporative water losses in the synthesis protocol, whereas batch 2 did consider evaporative water loss.

### Variation in Temperature-Dependence


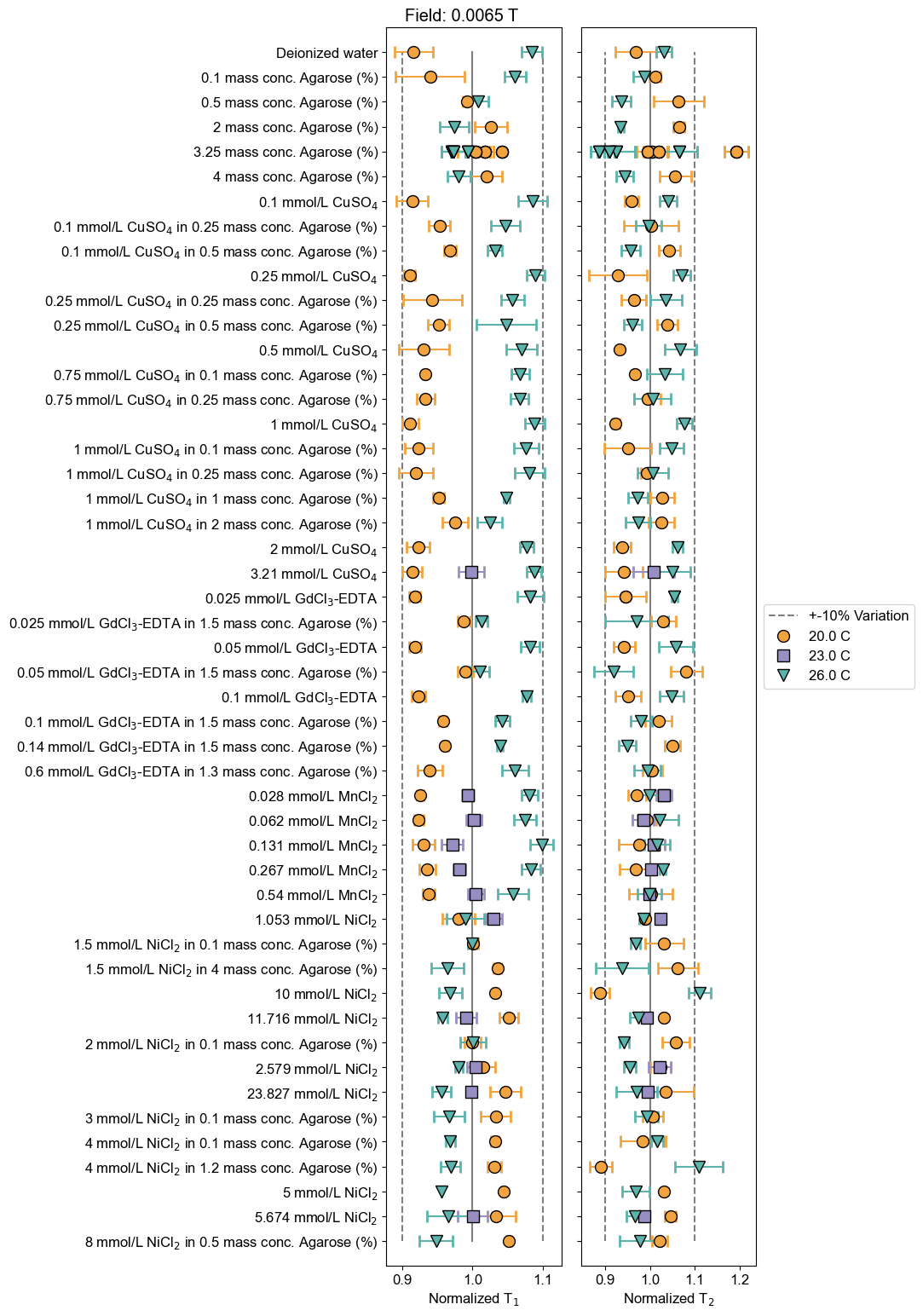


**Figure S11** Normalized $T_{1}$ (left) and $T_{2}$ (right) variation for a range of temperatures for 49 samples at 0.0065 T. Data are normalized to the average of the mean values for each sample. The measurement temperature is indicated by the marker.


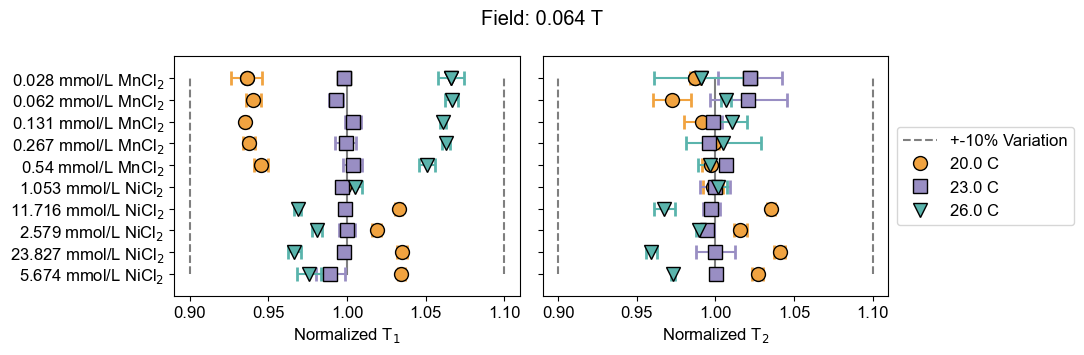


**Figure S12** Normalized $T_{1}$ (left) and $T_{2}$ (right) variation for a range of temperatures for 10 samples at 0.064 T. Data are normalized to the average of the mean values for each sample. The measurement temperature is indicated by the marker.


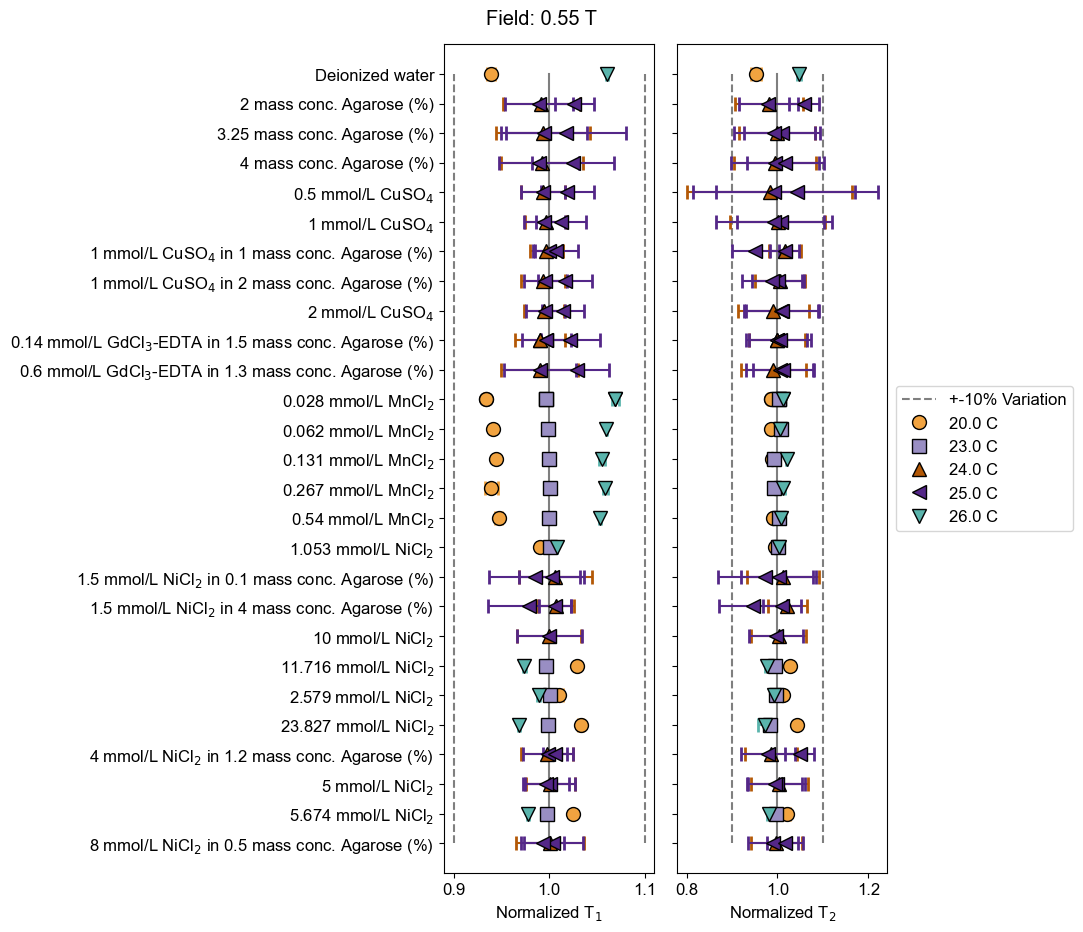


**Figure S13** Normalized $T_{1}$ (left) and $T_{2}$ (right) variation for a range of temperatures for 27 samples at 0.55 T. Data are normalized to the average of the mean values for each sample. The measurement temperature is indicated by the marker.

## Proof of Concept: Tissue Mimics using Mixing Model Recipes


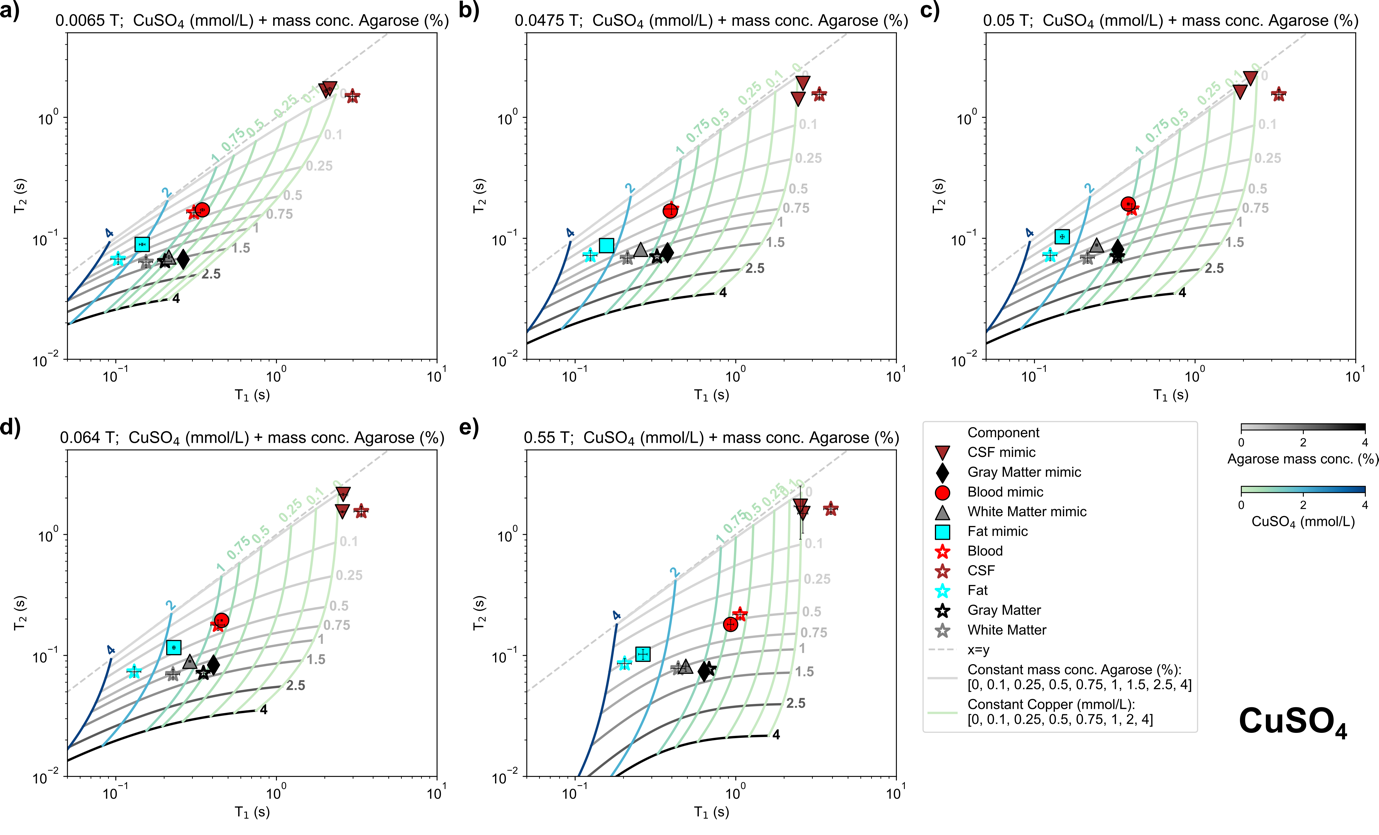


**Figure S14** $T_{1}$ and $T_{2}$ mimic sample measurements and mixing models for CuSO_4_ with agarose for (a) 0.0065 T, (b) 0.0475 T, (c) 0.05 T, (d) 0.064 T, (e) 0.55 T. Target tissue $T_{1}$ and $T_{2}$ times (stars; Blood=red, CSF=maroon, Fat=blue, GM=black, WM=gray) are shown. Each mimic measurement is shown with the same color as its tissue, and twice the standard deviation is plotted as error bars. Mixing models are displayed via constant agarose concentration lines (gray) and constant CuSO_4_ concentration lines (blue-green). Dashed gray line represents $T_{1}=T_{2}$.


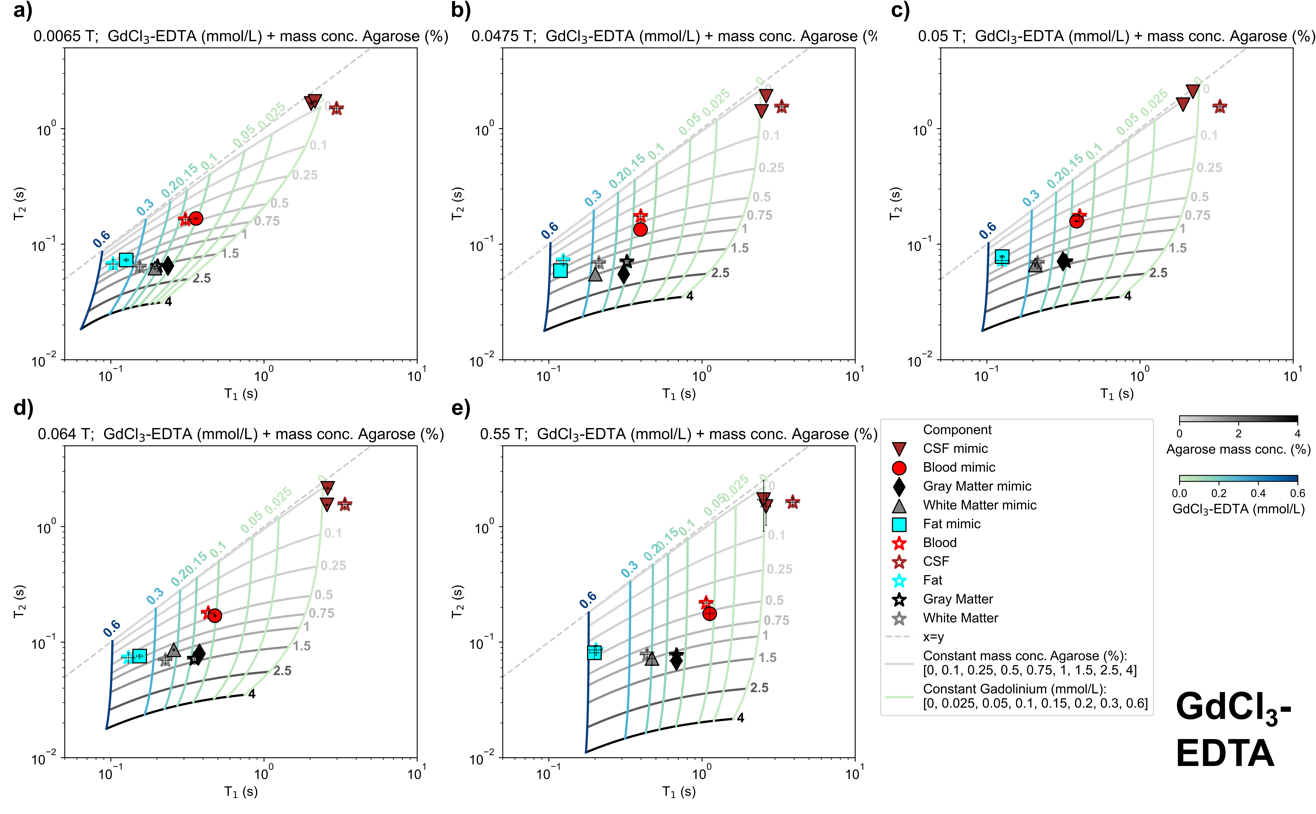


**Figure S15** $T_{1}$ and $T_{2}$ mimic sample measurements and mixing models for GdCl_3_-EDTA with agarose for (a) 0.0065 T, (b) 0.0475 T, (c) 0.05 T, (d) 0.064 T, (e) 0.55 T. Target tissue $T_{1}$ and $T_{2}$ times (stars; Blood=red, CSF=maroon, Fat=blue, GM=black, WM=gray) are shown. Each mimic measurement is shown with the same color as its tissue, and twice the standard deviation is plotted as error bars. Mixing models are displayed via constant agarose concentration lines (gray) and constant GdCl_3_-EDTA concentration lines (blue-green). Dashed gray line represents $T_{1}=T_{2}$.


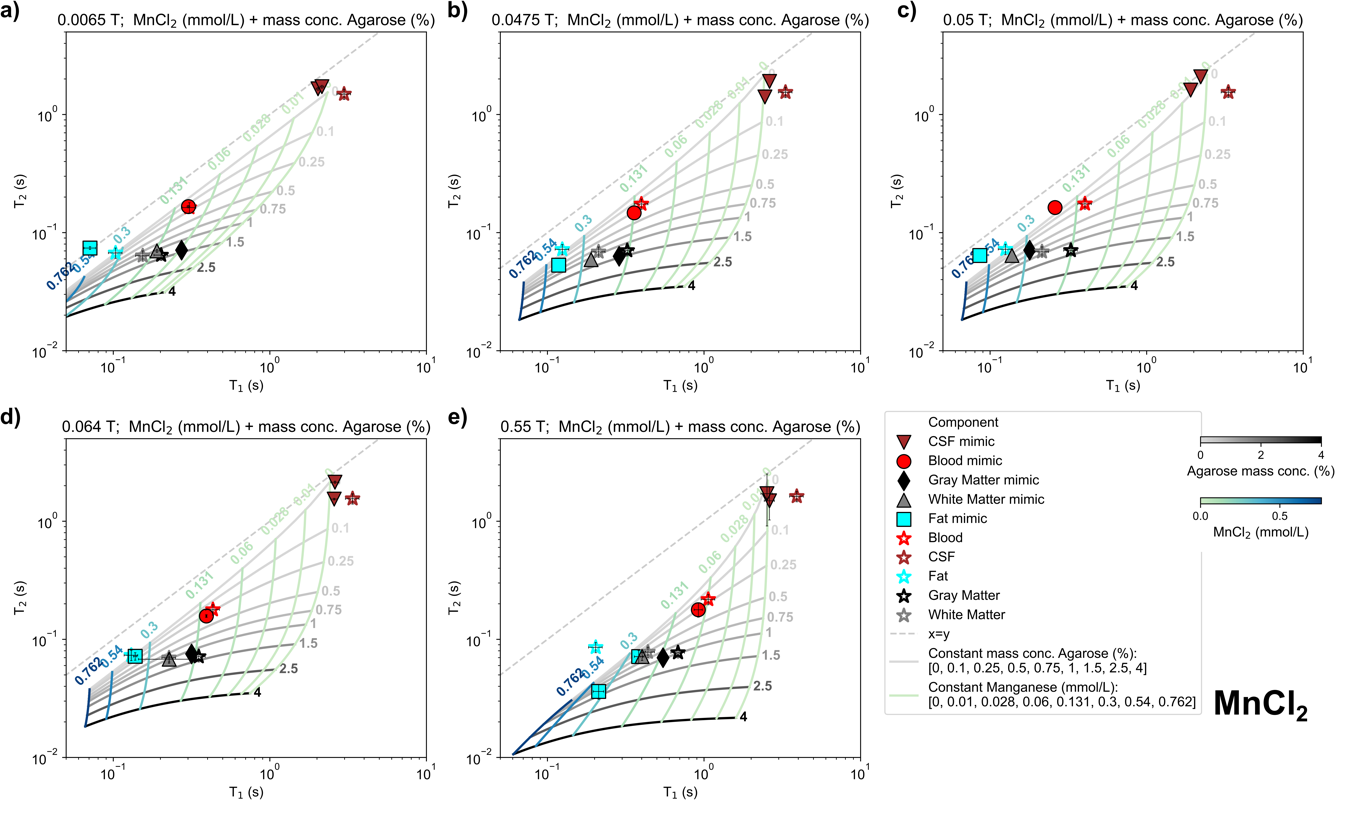


**Figure S16** $T_{1}$ and $T_{2}$ mimic sample measurements and mixing models for MnCl_2_ with agarose for (a) 0.0065 T, (b) 0.0475 T, (c) 0.05 T, (d) 0.064 T, (e) 0.55 T. Target tissue $T_{1}$ and $T_{2}$ times (stars; Blood=red, CSF=maroon, Fat=blue, GM=black, WM=gray) are shown. Each mimic measurement is shown with the same color as its tissue, and twice the standard deviation is plotted as error bars. Mixing models are displayed via constant agarose concentration lines (gray) and constant MnCl_2_ concentration lines (blue-green). Dashed gray line represents $T_{1}=T_{2}$.

The plot for the NiCl_2_ mimic sample measurements and mixing models is included as Figure 1 in the main manuscript.

|  |  |  |  | **T1** | | | **T2** | | |
| --- | --- | --- | --- | --- | --- | --- | --- | --- | --- |
| **Tissue** | **Field (T)** | **CuSO_4_ (mmol/L)** | **Agarose (mass conc. (%))** | **Target (s)** | **Measured (s)** | **Error (%)** | **Target (s)** | **Measured (s)** | **Error (%)** |
| Blood | 0.0065 | 1.014 | 0.37 | 0.306 | 0.346 | 13.1 | 0.163 | 0.172 | 5.5 |
|  | 0.0475 | 0.983 | 0.356 | 0.398 | 0.391 | -1.8 | 0.175 | 0.168 | -4 |
|  | 0.05 | 0.967 | 0.359 | 0.403 | 0.384 | -4.7 | 0.175 | 0.192 | 9.7 |
|  | 0.064 | 0.889 | 0.373 | 0.433 | 0.456 | 5.4 | 0.178 | 0.195 | 9.5 |
|  | 0.55 | 0.59 | 0.453 | 1.064 | 0.929 | -12.7 | 0.218 | 0.18 | -17.2 |
| CSF | 0.0065 | 0 | 0.003 | 2.978 | 2.03 | -31.8 | 1.49 | 1.65 | 10.7 |
|  | 0.0065 | 0 | 0 | 2.978 | 2.15 | -27.8 | 1.49 | 1.72 | 15.4 |
|  | 0.0475 | 0 | 0.027 | 3.315 | 2.449 | -26.1 | 1.543 | 1.408 | -8.7 |
|  | 0.0475 | 0 | 0 | 3.315 | 2.622 | -20.9 | 1.543 | 1.905 | 23.5 |
|  | 0.05 | 0 | 0.026 | 3.325 | 1.91 | -42.6 | 1.545 | 1.61 | 4.2 |
|  | 0.05 | 0 | 0 | 3.325 | 2.215 | -33.4 | 1.545 | 2.085 | 35 |
|  | 0.064 | 0 | 0.026 | 3.375 | 2.576 | -23.7 | 1.552 | 1.539 | -0.8 |
|  | 0.064 | 0 | 0 | 3.375 | 2.605 | -22.8 | 1.552 | 2.14 | 37.9 |
|  | 0.55 | 0 | 0.022 | 3.912 | 2.619 | -33.1 | 1.623 | 1.489 | -8.2 |
|  | 0.55 | 0 | 0 | 3.912 | 2.524 | -35.5 | 1.623 | 1.712 | 5.5 |
| Fat | 0.0065 | 2.729 | 0.637 | 0.103 | 0.146 | 41.7 | 0.067 | 0.089 | 32.8 |
|  | 0.0475 | 2.852 | 0.454 | 0.124 | 0.157 | 26.6 | 0.072 | 0.087 | 20.8 |
|  | 0.05 | 2.832 | 0.458 | 0.125 | 0.149 | 19.2 | 0.072 | 0.103 | 43.1 |
|  | 0.064 | 2.729 | 0.474 | 0.13 | 0.23 | 76.8 | 0.073 | 0.116 | 59.1 |
|  | 0.55 | 3.375 | 0.516 | 0.203 | 0.265 | 30.4 | 0.086 | 0.102 | 19 |
| GM | 0.0065 | 0.794 | 1.462 | 0.202 | 0.263 | 30.2 | 0.064 | 0.067 | 4.7 |
|  | 0.0475 | 0.87 | 1.279 | 0.323 | 0.376 | 16.4 | 0.071 | 0.076 | 7 |
|  | 0.05 | 0.854 | 1.285 | 0.328 | 0.33 | 0.6 | 0.071 | 0.081 | 14.1 |
|  | 0.064 | 0.779 | 1.315 | 0.351 | 0.406 | 15.6 | 0.071 | 0.083 | 17.4 |
|  | 0.55 | 0.876 | 1.21 | 0.682 | 0.635 | -6.9 | 0.077 | 0.074 | -4.5 |
| WM | 0.0065 | 1.333 | 1.229 | 0.154 | 0.214 | 39 | 0.064 | 0.07 | 9.4 |
|  | 0.0475 | 1.509 | 0.984 | 0.212 | 0.256 | 20.8 | 0.069 | 0.081 | 17.4 |
|  | 0.05 | 1.49 | 0.99 | 0.215 | 0.244 | 13.5 | 0.069 | 0.088 | 27.5 |
|  | 0.064 | 1.394 | 1.018 | 0.227 | 0.289 | 27.3 | 0.07 | 0.089 | 27.7 |
|  | 0.55 | 1.528 | 0.999 | 0.438 | 0.489 | 11.6 | 0.078 | 0.081 | 4.5 |

**Table S5** Target tissue T1 and T2 times for CuSO_4_ and agarose tissue mimics, along with CuSO_4_ and agarose concentrations. Measurements for T1 and T2 for each mimic sample at each field strength are listed, as well as the error of the measurement from both the target relaxation times.

|  |  |  |  | **T1** | | | **T2** | | |
| --- | --- | --- | --- | --- | --- | --- | --- | --- | --- |
| **Tissue** | **Field (T)** | **GdCl_3_ -EDTA (mmol/L)** | **Agarose (mass conc. (%))** | **Target (s)** | **Measured (s)** | **Error (%)** | **Target (s)** | **Measured (s)** | **Error (%)** |
| Blood | 0.0065 | 0.133 | 0.39 | 0.306 | 0.36 | 17.6 | 0.163 | 0.167 | 2.5 |
|  | 0.0475 | 0.13 | 0.403 | 0.398 | 0.397 | -0.3 | 0.175 | 0.134 | -23.4 |
|  | 0.05 | 0.128 | 0.406 | 0.403 | 0.385 | -4.5 | 0.175 | 0.158 | -9.7 |
|  | 0.064 | 0.117 | 0.419 | 0.433 | 0.478 | 10.4 | 0.178 | 0.169 | -4.9 |
|  | 0.55 | 0.063 | 0.405 | 1.064 | 1.123 | 5.5 | 0.218 | 0.176 | -19.1 |
| CSF | 0.0065 | 0 | 0.003 | 2.978 | 2.03 | -31.8 | 1.49 | 1.65 | 10.7 |
|  | 0.0065 | 0 | 0 | 2.978 | 2.15 | -27.8 | 1.49 | 1.72 | 15.4 |
|  | 0.0475 | 0 | 0.027 | 3.315 | 2.449 | -26.1 | 1.543 | 1.408 | -8.7 |
|  | 0.0475 | 0 | 0 | 3.315 | 2.622 | -20.9 | 1.543 | 1.905 | 23.5 |
|  | 0.05 | 0 | 0.026 | 3.325 | 1.91 | -42.6 | 1.545 | 1.61 | 4.2 |
|  | 0.05 | 0 | 0 | 3.325 | 2.215 | -33.4 | 1.545 | 2.085 | 35 |
|  | 0.064 | 0 | 0.026 | 3.375 | 2.576 | -23.7 | 1.552 | 1.539 | -0.8 |
|  | 0.064 | 0 | 0 | 3.375 | 2.605 | -22.8 | 1.552 | 2.14 | 37.9 |
|  | 0.55 | 0 | 0.022 | 3.912 | 2.619 | -33.1 | 1.623 | 1.489 | -8.2 |
|  | 0.55 | 0 | 0 | 3.912 | 2.524 | -35.5 | 1.623 | 1.712 | 5.5 |
| Fat | 0.0065 | 0.472 | 0.587 | 0.103 | 0.126 | 22.3 | 0.067 | 0.073 | 9 |
|  | 0.0475 | 0.484 | 0.549 | 0.124 | 0.119 | -4 | 0.072 | 0.059 | -18.1 |
|  | 0.05 | 0.48 | 0.553 | 0.125 | 0.126 | 0.8 | 0.072 | 0.078 | 8.3 |
|  | 0.064 | 0.46 | 0.572 | 0.13 | 0.154 | 18.7 | 0.073 | 0.076 | 3.8 |
|  | 0.55 | 0.531 | 0.409 | 0.203 | 0.198 | -2.4 | 0.086 | 0.081 | -6 |
| GM | 0.0065 | 0.16 | 1.504 | 0.202 | 0.236 | 16.8 | 0.064 | 0.065 | 1.6 |
|  | 0.0475 | 0.156 | 1.376 | 0.323 | 0.308 | -4.6 | 0.071 | 0.055 | -22.5 |
|  | 0.05 | 0.153 | 1.381 | 0.328 | 0.314 | -4.3 | 0.071 | 0.071 | 0 |
|  | 0.064 | 0.14 | 1.405 | 0.351 | 0.379 | 8 | 0.071 | 0.079 | 11.8 |
|  | 0.55 | 0.121 | 1.091 | 0.682 | 0.681 | -0.2 | 0.077 | 0.069 | -10.4 |
| WM | 0.0065 | 0.259 | 1.25 | 0.154 | 0.194 | 26 | 0.064 | 0.062 | -3.1 |
|  | 0.0475 | 0.263 | 1.107 | 0.212 | 0.201 | -5.2 | 0.069 | 0.055 | -20.3 |
|  | 0.05 | 0.26 | 1.112 | 0.215 | 0.207 | -3.7 | 0.069 | 0.066 | -4.3 |
|  | 0.064 | 0.243 | 1.138 | 0.227 | 0.258 | 13.5 | 0.07 | 0.086 | 22.5 |
|  | 0.55 | 0.218 | 0.885 | 0.438 | 0.471 | 7.6 | 0.078 | 0.072 | -7.5 |

**Table S6** Target tissue T1 and T2 times for GdCl_3_-EDTA and agarose tissue mimics, along with GdCl_3_-EDTA and agarose concentrations. Measurements for T1 and T2 for each mimic sample at each field strength are listed, as well as the error of the measurement from both the target relaxation times.

|  |  |  |  | **T1** | | | **T2** | | |
| --- | --- | --- | --- | --- | --- | --- | --- | --- | --- |
| **Tissue** | **Field (T)** | **MnCl_2_ (mmol/L)** | **Agarose (mass conc. (%))** | **Target (s)** | **Measured (s)** | **Error (%)** | **Target (s)** | **Measured (s)** | **Error (%)** |
| Blood | 0.0065 | 0.095 | 0.184 | 0.306 | 0.302 | -1.3 | 0.163 | 0.166 | 1.8 |
|  | 0.0475 | 0.115 | 0.192 | 0.398 | 0.358 | -10.1 | 0.175 | 0.147 | -16 |
|  | 0.05 | 0.113 | 0.198 | 0.403 | 0.26 | -35.5 | 0.175 | 0.163 | -6.9 |
|  | 0.064 | 0.103 | 0.23 | 0.433 | 0.393 | -9.2 | 0.178 | 0.158 | -11.5 |
|  | 0.55 | 0.059 | 0.205 | 1.064 | 0.92 | -13.5 | 0.218 | 0.178 | -18.3 |
| CSF | 0.0065 | 0 | 0.003 | 2.978 | 2.03 | -31.8 | 1.49 | 1.65 | 10.7 |
|  | 0.0065 | 0 | 0 | 2.978 | 2.15 | -27.8 | 1.49 | 1.72 | 15.4 |
|  | 0.0475 | 0 | 0.027 | 3.315 | 2.449 | -26.1 | 1.543 | 1.408 | -8.7 |
|  | 0.0475 | 0 | 0 | 3.315 | 2.622 | -20.9 | 1.543 | 1.905 | 23.5 |
|  | 0.05 | 0 | 0.026 | 3.325 | 1.91 | -42.6 | 1.545 | 1.61 | 4.2 |
|  | 0.05 | 0 | 0 | 3.325 | 2.215 | -33.4 | 1.545 | 2.085 | 35 |
|  | 0.064 | 0 | 0.026 | 3.375 | 2.576 | -23.7 | 1.552 | 1.539 | -0.8 |
|  | 0.064 | 0 | 0 | 3.375 | 2.605 | -22.8 | 1.552 | 2.14 | 37.9 |
|  | 0.55 | 0 | 0.022 | 3.912 | 2.619 | -33.1 | 1.623 | 1.489 | -8.2 |
|  | 0.55 | 0 | 0 | 3.912 | 2.524 | -35.5 | 1.623 | 1.712 | 5.5 |
| Fat | 0.0065 | 0.336 | 0 | 0.103 | 0.071 | -31.1 | 0.067 | 0.074 | 10.4 |
|  | 0.0475 | 0.424 | 0 | 0.124 | 0.118 | -4.8 | 0.072 | 0.053 | -26.4 |
|  | 0.05 | 0.42 | 0 | 0.125 | 0.086 | -31.2 | 0.072 | 0.064 | -11.1 |
|  | 0.064 | 0.403 | 0 | 0.13 | 0.138 | 6.2 | 0.073 | 0.072 | -1.6 |
|  | 0.55 | 0.264 | 0 | 0.203 | 0.383 | 88.5 | 0.086 | 0.071 | -16.9 |
|  | 0.55 | 0.521 | 0 | 0.203 | 0.212 | 4.7 | 0.086 | 0.036 | -57.9 |
| GM | 0.0065 | 0.1 | 1.325 | 0.202 | 0.273 | 35.1 | 0.064 | 0.071 | 10.9 |
|  | 0.0475 | 0.135 | 1.294 | 0.323 | 0.287 | -11.1 | 0.071 | 0.063 | -11.3 |
|  | 0.05 | 0.133 | 1.301 | 0.328 | 0.179 | -45.4 | 0.071 | 0.071 | 0 |
|  | 0.064 | 0.121 | 1.336 | 0.351 | 0.316 | -9.9 | 0.071 | 0.075 | 5.9 |
|  | 0.55 | 0.09 | 0.976 | 0.682 | 0.547 | -19.7 | 0.077 | 0.07 | -9.2 |
| WM | 0.0065 | 0.168 | 0.971 | 0.154 | 0.19 | 23.4 | 0.064 | 0.07 | 9.4 |
|  | 0.0475 | 0.23 | 0.888 | 0.212 | 0.19 | -10.4 | 0.069 | 0.059 | -14.5 |
|  | 0.05 | 0.227 | 0.898 | 0.215 | 0.138 | -35.8 | 0.069 | 0.064 | -7.2 |
|  | 0.064 | 0.212 | 0.945 | 0.227 | 0.228 | 0.3 | 0.07 | 0.068 | -3 |
|  | 0.55 | 0.185 | 0.506 | 0.438 | 0.402 | -8.2 | 0.078 | 0.072 | -8.2 |

**Table S7** Target tissue T1 and T2 times for MnCl_2_ and agarose tissue mimics, along with MnCl_2_ and agarose concentrations. Measurements for T1 and T2 for each mimic sample at each field strength are listed, as well as the error of the measurement from both the target relaxation times.

|  |  |  |  | **T1** |  |  | **T2** |  |  |
| --- | --- | --- | --- | --- | --- | --- | --- | --- | --- |
| **Tissue** | **Field (T)** | **NiCl_2_ (mmol/L)** | **Agarose (mass conc. (%))** | **Target (s)** | **Measured (s)** | **Error (%)** | **Target (s)** | **Measured (s)** | **Error (%)** |
| Blood | 0.0065 | 4.497 | 0.322 | 0.306 | 0.324 | 5.9 | 0.163 | 0.18 | 10.4 |
|  | 0.0475 | 3.563 | 0.457 | 0.398 | 0.424 | 6.5 | 0.175 | 0.158 | -9.7 |
|  | 0.05 | 3.504 | 0.459 | 0.403 | 0.325 | -19.4 | 0.175 | 0.178 | 1.7 |
|  | 0.064 | 3.204 | 0.469 | 0.433 | 0.432 | -0.3 | 0.178 | 0.174 | -2.1 |
|  | 0.55 | 0.816 | 0.453 | 1.064 | 1.104 | 3.8 | 0.218 | 0.197 | -9.7 |
| CSF | 0.0065 | 0 | 0.003 | 2.978 | 2.03 | -31.8 | 1.49 | 1.65 | 10.7 |
|  | 0.0065 | 0 | 0 | 2.978 | 2.15 | -27.8 | 1.49 | 1.72 | 15.4 |
|  | 0.0475 | 0 | 0.027 | 3.315 | 2.449 | -26.1 | 1.543 | 1.408 | -8.7 |
|  | 0.0475 | 0 | 0 | 3.315 | 2.622 | -20.9 | 1.543 | 1.905 | 23.5 |
|  | 0.05 | 0 | 0.026 | 3.325 | 1.91 | -42.6 | 1.545 | 1.61 | 4.2 |
|  | 0.05 | 0 | 0 | 3.325 | 2.215 | -33.4 | 1.545 | 2.085 | 35 |
|  | 0.064 | 0 | 0.026 | 3.375 | 2.576 | -23.7 | 1.552 | 1.539 | -0.8 |
|  | 0.064 | 0 | 0 | 3.375 | 2.605 | -22.8 | 1.552 | 2.14 | 37.9 |
|  | 0.55 | 0 | 0.022 | 3.912 | 2.619 | -33.1 | 1.623 | 1.489 | -8.2 |
|  | 0.55 | 0 | 0 | 3.912 | 2.524 | -35.5 | 1.623 | 1.712 | 5.5 |
| Fat | 0.0065 | 15.65 | 0.349 | 0.103 | 0.118 | 14.6 | 0.067 | 0.087 | 29.9 |
|  | 0.0475 | 13.201 | 0.747 | 0.124 | 0.126 | 1.6 | 0.072 | 0.066 | -8.3 |
|  | 0.05 | 13.093 | 0.751 | 0.125 | 0.107 | -14.4 | 0.072 | 0.079 | 9.7 |
|  | 0.064 | 12.551 | 0.773 | 0.13 | 0.142 | 9 | 0.073 | 0.075 | 2.3 |
|  | 0.55 | 6.834 | 0.638 | 0.203 | 0.222 | 9.2 | 0.086 | 0.091 | 5.4 |
| GM | 0.0065 | 5.915 | 1.152 | 0.202 | 0.203 | 0.5 | 0.064 | 0.08 | 25 |
|  | 0.0475 | 4.193 | 1.593 | 0.323 | 0.334 | 3.4 | 0.071 | 0.065 | -8.5 |
|  | 0.05 | 4.116 | 1.596 | 0.328 | 0.258 | -21.3 | 0.071 | 0.074 | 4.2 |
|  | 0.064 | 3.756 | 1.607 | 0.351 | 0.366 | 4.3 | 0.071 | 0.072 | 2.1 |
|  | 0.55 | 1.564 | 1.293 | 0.682 | 0.682 | 0 | 0.077 | 0.075 | -2.5 |
| WM | 0.0065 | 9.229 | 0.835 | 0.154 | 0.173 | 12.3 | 0.064 | 0.084 | 31.3 |
|  | 0.0475 | 7.129 | 1.379 | 0.212 | 0.221 | 4.2 | 0.069 | 0.063 | -8.7 |
|  | 0.05 | 7.034 | 1.383 | 0.215 | 0.169 | -21.4 | 0.069 | 0.073 | 5.8 |
|  | 0.064 | 6.574 | 1.401 | 0.227 | 0.237 | 4.6 | 0.07 | 0.076 | 8.6 |
|  | 0.55 | 2.816 | 1.164 | 0.438 | 0.487 | 11.1 | 0.078 | 0.076 | -2.1 |

**Table S8** Target tissue T1 and T2 times for NiCl_2_ and agarose tissue mimics, along with NiCl_2_ and agarose concentrations. Measurements for T1 and T2 for each mimic sample at each field strength are listed, as well as the error of the measurement from both the target relaxation times.

# References

1. Bernstein, Matt A, King, Kevin F, Zhou, Xiaohong Joe. Basic Pulse Sequences - Inversion Recovery. In: *Handbook of MRI Pulse Sequences*. 1st Edition. Elsevier; 2004:579-647. Accessed November 29, 2022. https://www.elsevier.com/books/handbook-of-mri-pulse-sequences/bernstein/978-0-12-092861-3

2. Anoardo E, Galli G, Ferrante G. Fast-field-cycling NMR: Applications and instrumentation. *Appl Magn Reson*. 2001;20(3):365-404. doi:10.1007/BF03162287

3. Kimmich R, Anoardo E. Field-Cycling NMR Relaxometry. *ChemInform*. 2004;35(47). doi:10.1002/chin.200447278

4. Rohatgi, Ankit. Webplotdigitizer. Published online 2022. https://automeris.io/WebPlotDigitizer

5. Broche LM, Ross PJ, Davies GR, MacLeod MJ, Lurie DJ. A whole-body Fast Field-Cycling scanner for clinical molecular imaging studies. *Sci Rep*. 2019;9(1):10402. doi:10.1038/s41598-019-46648-0

6. Mitchell MD, Kundel HL, Axel L, Joseph PM. Agarose as a tissue equivalent phantom material for NMR imaging. *Magn Reson Imaging*. 1986;4(3):263-266. doi:10.1016/0730-725X(86)91068-4

7. Christoffersson JO, Olsson LE, Sjöberg S. Nickel-Doped Agarose Gel Phantoms in MR Imaging. *Acta Rudiologica*. 1991;32(5):426-431.

8. Hartsgrove G, Kraszewski A, Surowiec A. Simulated biological materials for electromagnetic radiation absorption studies. *Bioelectromagnetics*. 1987;8(1):29-36. doi:10.1002/bem.2250080105

9. Koenig SH, Brown RD. Determinants of Proton Relaxation Rates in Tissue. *Magn Reson Med*. 1984;1(4):437-449. doi:10.1002/mrm.1910010404

10. Lacomis D, Osbakken M, Gross G. Spin-lattice relaxation (T1) times of cerebral white matter in multiple sclerosis. *Magn Reson Med*. 1986;3(2):194-202. doi:10.1002/mrm.1910030203

11. Hopkins AL, Yeung HN, Bratton CB. Multiple field strength in vivo T1 and T2 for cerebrospinal fluid protons. *Magn Reson Med*. 1986;3(2):303-311. doi:10.1002/mrm.1910030214

12. Breger RK, Wehrli FW, Charles HC, MacFall JR, Haughton VM. Reproducibility of relaxation and spin-density parameters in phantoms and the human brain measured by MR imaging at 1.5 T. *Magn Reson Med*. 1986;3(5):649-662. doi:10.1002/mrm.1910030502

13. Mano I, Goshima H, Nambu M, Iio M. New polyvinyl alcohol gel material for MRI phantoms. *Magn Reson Med*. 1986;3(6):921-926. doi:10.1002/mrm.1910030612

14. Kraft KA, Fatouros PP, Clarke GD, Kishore PRS. An MRI phantom material for quantitative relaxometry. *Magn Reson Med*. 1987;5(6):555-562. doi:10.1002/mrm.1910050606

15. Santyr GE, Henkelman RM, Bronskill MJ. Spin locking for magnetic resonance imaging with application to human breast. *Magn Reson Med*. 1989;12(1):25-37. doi:10.1002/mrm.1910120104

16. Koenig SH, Brown RD, Spiller M, Lundbom N. Relaxometry of brain: Why white matter appears bright in MRI. *Magn Reson Med*. 1990;14(3):482-495. doi:10.1002/mrm.1910140306

17. Fischer HW, Rinck PA, Van Haverbeke Y, Muller RN. Nuclear relaxation of human brain gray and white matter: analysis of field dependence and implications for MRI. *Magn Reson Med*. 1990;16(2):317-334. doi:10.1002/mrm.1910160212

18. Lee SK, Mössle M, Myers W, et al. SQUID-detected MRI at 132 microT with T1-weighted contrast established at 10 microT--300 mT. *Magn Reson Med*. 2005;53(1):9-14. doi:10.1002/mrm.20316

19. Stanisz GJ, Odrobina EE, Pun J, et al. T1, T2 relaxation and magnetization transfer in tissue at 3T. *Magn Reson Med*. 2005;54(3):507-512. doi:10.1002/mrm.20605

20. Rooney WD, Johnson G, Li X, et al. Magnetic field and tissue dependencies of human brain longitudinal1H2O relaxation in vivo. *Magn Reson Med*. 2007;57(2):308-318. doi:10.1002/mrm.21122

21. Zhang X, Petersen ET, Ghariq E, et al. In vivo blood T1 measurements at 1.5 T, 3 T, and 7 T. *Magn Reson Med*. 2013;70(4):1082-1086. doi:10.1002/mrm.24550

22. Krishnamurthy LC, Liu P, Xu F, Uh J, Dimitrov I, Lu H. Dependence of blood T2 on oxygenation at 7 T: In vitro calibration and in vivo application. *Magn Reson Med*. 2014;71(6):2035-2042. doi:10.1002/mrm.24868

23. Pohmann R, Speck O, Scheffler K. Signal‐to‐noise ratio and MR tissue parameters in human brain imaging at 3, 7, and 9.4 tesla using current receive coil arrays. *Magn Reson Med*. 2016;75(2):801-809. doi:10.1002/mrm.25677

24. Bödenler M, Maier O, Stollberger R, et al. Joint multi-field T1 quantification for fast field-cycling MRI. *Magn Reson Med*. 2021;86(4):2049-2063. doi:10.1002/mrm.28857

25. Koenig SH, Brown RD. Field-cycling relaxometry of protein solutions and tissue: Implications for MRI. *Prog Nucl Magn Reson Spectrosc*. 1990;22(6):487-567. doi:10.1016/0079-6565(90)80008-6

26. Madsen EL, Fullerton GD. Prospective tissue-mimicking materials for use in NMR imaging phantoms. *Magn Reson Imaging*. 1982;1(3):135-141. doi:10.1016/0730-725X(82)90204-1

27. Mendelson DA, Heinsbergen JF, Kennedy SD, Szczepaniak LS, Lester CC, Bryant RG. Comparison of agarose and cross-linked protein gels as magnetic resonance imaging phantoms. *Magn Reson Imaging*. 1991;9(6):975-978. doi:10.1016/0730-725X(91)90546-X

28. Lurie DJ, Aime S, Baroni S, et al. Fast field-cycling magnetic resonance imaging. *Comptes Rendus Phys*. 2010;11(2):136-148. doi:10.1016/j.crhy.2010.06.012

29. Baranowska H, Sikora M, Kowalski S, Tomasik P. Interactions of potato starch with selected polysaccharide hydrocolloids as measured by low-field NMR. *Food Hydrocoll*. 2008;22(2):336-345. doi:10.1016/j.foodhyd.2006.12.014

30. Wu J, Li L, Wu X, Dai Q, Zhang R, Zhang Y. Characterization of Oat ( *Avena nuda* L.) β-Glucan Cryogelation Process by Low-Field NMR. *J Agric Food Chem*. 2016;64(1):310-319. doi:10.1021/acs.jafc.5b03948

31. Masiewicz E, Ashcroft GP, Boddie D, Dundas SR, Kruk D, Broche LM. Towards applying NMR relaxometry as a diagnostic tool for bone and soft tissue sarcomas: a pilot study. *Sci Rep*. 2020;10(1):14207. doi:10.1038/s41598-020-71067-x

32. Abrami M, D’Agostino I, Milcovich G, et al. Physical characterization of alginate–Pluronic F127 gel for endoluminal NABDs delivery. *Soft Matter*. 2014;10(5):729-737. doi:10.1039/C3SM51873F

33. Fiorentino SM, Carfì Pavia F, La Carrubba V, et al. Characterization of PLLA scaffolds for biomedical applications. *Int J Polym Mater Polym Biomater*. 2017;66(9):469-477. doi:10.1080/00914037.2016.1252344

34. Walker PM, Balmer C, Ablett S, Lerski RA. A test material for tissue characterisation and system calibration in MRI. *Phys Med Biol*. 1989;34(1):5-22. doi:10.1088/0031-9155/34/1/002

35. Ponsiglione AM, Russo M, Netti PA, Torino E. Impact of biopolymer matrices on relaxometric properties of contrast agents. *Interface Focus*. 2016;6(6):20160061. doi:10.1098/rsfs.2016.0061

36. Zotev VS, Matlashov AN, Savukov IM, et al. SQUID-Based Microtesla MRI for In Vivo Relaxometry of the Human Brain. *IEEE Trans Appl Supercond*. 2009;19(3):823-826. doi:10.1109/TASC.2009.2018764

37. Bottomley PA, Foster TH, Argersinger RE, Pfeifer LM. A review of normal tissue hydrogen NMR relaxation times and relaxation mechanisms from 1-100 MHz: dependence on tissue type, NMR frequency, temperature, species, excision, and age. *Med Phys*. 1984;11(4):425-448. doi:10.1118/1.595535

38. Campbell-Washburn AE, Ramasawmy R, Restivo MC, et al. Opportunities in Interventional and Diagnostic Imaging by Using High-Performance Low-Field-Strength MRI. *Radiology*. 2019;293(2):384-393. doi:10.1148/radiol.2019190452

39. Araki T, Inouye T, Suzuki H, Machida T, Iio M. Magnetic resonance imaging of brain tumors: measurement of T1. Work in progress. *Radiology*. 1984;150(1):95-98. doi:10.1148/radiology.150.1.6689793

40. Just M, Thelen M. Tissue characterization with T1, T2, and proton density values: results in 160 patients with brain tumors. *Radiology*. 1988;169(3):779-785. doi:10.1148/radiology.169.3.3187000

41. van Leeuwen FHP, Lena B, Zwanenburg JJM, et al. Detecting low blood concentrations in joints using T1 and T2 mapping at 1.5, 3, and 7 T: an in vitro study. *Eur Radiol Exp*. 2021;5(1):51. doi:10.1186/s41747-021-00251-z

42. Bydder GM, Steiner RE, Young IR, et al. Clinical NMR imaging of the brain: 140 cases. *AJR Am J Roentgenol*. 1982;139(2):215-236. doi:10.2214/ajr.139.2.215

43. Komiyama M, Yagura H, Baba M, et al. MR imaging: possibility of tissue characterization of brain tumors using T1 and T2 values. *AJNR Am J Neuroradiol*. 1987;8(1):65-70.

44. O’Reilly T, Webb AG. In vivo T1 and T2 relaxation time maps of brain tissue, skeletal muscle, and lipid measured in healthy volunteers at 50 mT. *Magn Reson Med*. 2021;n/a(n/a). doi:10.1002/mrm.29009

45. Deoni SCL, O’Muircheartaigh J, Ljungberg E, Huentelman M, Williams SCR. Simultaneous high-resolution T2-weighted imaging and quantitative T2 mapping at low magnetic field strengths using a multiple TE and multi-orientation acquisition approach. *Magn Reson Med*. 2022;88(3):1273-1281. doi:10.1002/mrm.29273

46. Oros-Peusquens AM, Laurila M, Shah NJ. Magnetic field dependence of the distribution of NMR relaxation times in the living human brain. *Magma N Y N*. 2008;21(1-2):131-147. doi:10.1007/s10334-008-0107-5

47. Stupic KF, Ainslie M, Boss MA, et al. A standard system phantom for magnetic resonance imaging. *Magn Reson Med*. 2021;86(3):1194-1211. doi:10.1002/mrm.28779

48. Kjos B, Ehman R, Brant-Zawadzki M, Kelly W, Norman D, Newton T. Reproducibility of relaxation times and spin density calculated from routine MR imaging sequences: clinical study of the CNS. *Am J Roentgenol*. 1985;144(6):1165-1170. doi:10.2214/ajr.144.6.1165

49. Baas KPA, Coolen BF, Petersen ET, Biemond BJ, Strijkers GJ, Nederveen AJ. Comparative Analysis of Blood T2 Values Measured by T2-TRIR and TRUST. *J Magn Reson Imaging*. 2022;56(2):516-526. doi:10.1002/jmri.28066

50. Bloembergen N, Purcell EM, Pound RV. Relaxation Effects in Nuclear Magnetic Resonance Absorption. *Phys Rev*. 1948;73(7):679-712. doi:10.1103/PhysRev.73.679

51. Solomon I. Relaxation Processes in a System of Two Spins. *Phys Rev*. 1955;99(2):559-565. doi:10.1103/PhysRev.99.559

52. Halle B. Molecular theory of field-dependent proton spin-lattice relaxation in tissue. *Magn Reson Med*. 2006;56(1):60-72. doi:10.1002/mrm.20919

53. Bryant RG, Marill K, Blackmore C, Francis C. Magnetic relaxation in blood and blood clots. *Magn Reson Med*. 1990;13(1):133-144. doi:10.1002/mrm.1910130112

54. Kruk D, Masiewicz E, Borkowska AM, et al. Dynamics of Solid Proteins by Means of Nuclear Magnetic Resonance Relaxometry. *Biomolecules*. 2019;9(11):652. doi:10.3390/biom9110652

55. Boulby PA, Rugg–Gunn FJ. T2: The Transverse Relaxation Time. In: *Quantitative MRI of the Brain*. John Wiley & Sons, Ltd; 2003:143-201. doi:10.1002/0470869526.ch6

56. Lankhorst D, Schriever J, Leyte JC. Determination of the Rotational Correlation Time of Water by Proton NMR Relaxation in H217O and Some Related Results. *Berichte Bunsenges Für Phys Chem*. 1982;86(3):215-221. doi:10.1002/bbpc.19820860308

57. Mishra VK, Anantharamaiah GM, Segrest JP, et al. Association of a model class A (apolipoprotein) amphipathic alpha helical peptide with lipid: high resolution NMR studies of peptide.lipid discoidal complexes. *J Biol Chem*. 2006;281(10):6511-6519. doi:10.1074/jbc.M511475200
